# Biodiversity priority areas and conservation strategies for seed plants in China

**DOI:** 10.3389/fpls.2022.962609

**Published:** 2022-08-12

**Authors:** Xudong Yang, Wendi Zhang, Fei Qin, Jianghong Yu, Tiantian Xue, Yunfeng Huang, Weibin Xu, Jianyong Wu, Erik F. Smets, Shengxiang Yu

**Affiliations:** ^1^State Key Laboratory of Systematic and Evolutionary Botany, Institute of Botany, Chinese Academy of Sciences, Beijing, China; ^2^University of Chinese Academy of Sciences, Beijing, China; ^3^College of Forestry, Guizhou University, Guiyang, China; ^4^Guangxi Institute of Traditional Medical and Pharmaceutical Sciences, Nanning, China; ^5^Guangxi Key Laboratory of Plant Conservation and Restoration Ecology in Karst Terrain, Guangxi Institute of Botany, Guangxi Zhuangzu Autonomous Region and Chinese Academy of Sciences, Guilin, China; ^6^Centre for Biodiversity Conservation and Biosafety, Nanjing Institute of Environmental Sciences, Ministry of Ecology and Environment of China, Nanjing, China; ^7^Naturalis Biodiversity Centre, Leiden, Netherlands; ^8^Institute of Biology Leiden, Leiden University, Leiden, Netherlands; ^9^Ecology, Evolution and Biodiversity Conservation, KU Leuven, Leuven, Belgium

**Keywords:** biodiversity hotspot, complementary algorithm, correlation analysis, priority areas, phylogenetic diversity, species richness

## Abstract

China is known for its abundant plant resources, but biodiversity conservation faces unprecedented challenges. To provide feasible suggestions for sustainable conservation, we used the species richness algorithm and complementary algorithm to study distribution patterns of 34,082 seed plants based on 1,007,196 county-level distribution records. We reconstructed a phylogenetic tree for 95.35% of species and estimated the spatial phylogenetics, followed by correlation analyses between different distribution patterns. We identified 264 counties concentrated in southern and south-western mountainous areas as hotspots which covered 10% of the land area of China and harbored 85.22% of the Chinese seed plant species. The biodiversity conservation priorities we identified were highly representative as we have considered multiple conservation indicators. We evaluated the conservation effectiveness and gaps in the network of nature reserves and identified 31.44, 32.95, and 9.47%, respectively, of the hotspot counties as gaps in the national nature reserves, provincial nature reserves and both together, with respectively 55.77, 61.53, and 28.94% of the species. Analysis of the species composition showed there were a large number of threatened and endemic species occurring in the nature reserves’ gaps. The conservation gaps need to be filled by establishing new nature reserves or national parks, especially in south-western China, and more attentions should be paid to strengthen the conservation of specific plant taxa due to the apparent mismatches between different distribution patterns.

## Introduction

Human health and well-being rely on numerous ecosystem services provided by the wealth of biological diversity on our planet. However, population growth and urbanization have caused severe environmental problems, such as habitat loss and fragmentation, overutilization of biological resources, invasive species, pollution, and even climate change, which lead to the decline of biodiversity on a global scale (Williams et al., 2011; Ren and Duan, 2017). The Kunming Declaration made it clear that the loss of biodiversity and a series of serious environmental problems constitute unprecedented and interrelated crises that threaten the survival of our planet (Xi, 2021). More than 37,400 species around the world are threatened with extinction and 22–47% of the world’s plants are endangered (Pitman and Jørgensen, 2002; Monastersky, 2014; Teller et al., 2015; IUCN, 2021). As a result, biodiversity conservation has attracted great attention, in which the development of *in situ* conservation networks is crucial (Myers et al., 2000; Brooks et al., 2006; Huang et al., 2012; Wang and Li, 2021). Although protected areas have expanded greatly, they still provide low species coverage and are not sufficient to optimally conserve biodiversity (Pimm et al., 2014; Venter et al., 2014). Given the urgency, preserving biodiversity still faces unprecedented challenges (Zhang D. et al., 2016; Liang et al., 2018).

By identifying priority areas and gaps for conservation, resources can be used efficiently to maximize biodiversity conservation (Brooks et al., 2006; Huang et al., 2016; Zhang Y. et al., 2017). Identification of biodiversity hotspots, which are biogeographic areas with exceptionally high numbers of biodiversity, has an important role in biodiversity conservation (Myers et al., 2000). However, biodiversity hotspots are usually identified based on species (taxonomic) richness alone (Luo et al., 2016; Zhao et al., 2016; Brum et al., 2017), which overlooks other aspects of biodiversity (Huang et al., 2012; Chi et al., 2017). Hence, more comprehensive approaches should be applied to identify the most valuable areas to maintain biodiversity, regardless of whether they are “species-rich” (Marchese, 2015). Therefore, a complementary algorithm that emphasizes the irreplaceability of species has been used, providing a new perspective for biodiversity hotspot identification (Dobson et al., 1997; Zhang et al., 2015b; Chi et al., 2017). In recent years, the combination of spatial phylogenetics and geographical distribution patterns of regional plant species has also been applied in biodiversity conservation (Huang et al., 2016; Kougioumoutzis et al., 2020; Zhu et al., 2021). Phylogenetic diversity is a measure of biodiversity based on the evolutionary history (phylogeny) and together with functional diversity, which refers to the variety of growth forms and functional traits (Faith, 2013; Kelly et al., 2014; Quan et al., 2018; Le Bagousse-Pinguet et al., 2019), it can be used to identify priority areas for conservation (Huang et al., 2016).

We therefore believe that it is necessary to identify biodiversity priority areas based on more comprehensive approaches in order to provide scientific guidance for establishing new protected areas, adjusting the existing conservation system, and developing effective protection measures (Luo et al., 2016; Zhang Y. et al., 2017; Volis, 2018). To evaluate the status of the biodiversity hotspots in China, most studies have focused on several particular groups of plant species, such as endemic species (Huang et al., 2016), threatened species (Zhang et al., 2015a), medicinal plants (Chi et al., 2017), and several crucial taxa. Unfortunately, the spatial patterns of species richness among all species, endemic species and threatened species are often inconsistent with each other (Orme et al., 2005; Tang et al., 2006).

China covers a vast geographical area of 9,600,000 km^2^ with many mountains systems with a wide range of elevations (Supplementary Figure 1), and the country contains almost all of the biomes found on Earth (Tang et al., 2006). This geographic diversity provides abundant habitats for a wide range of plant species. About 35,000 species of higher plants grow in China, accounting for about 10% of the world’s higher plants (Xie et al., 2021), which means that China is one of the countries with the richest plant diversity in the world. In addition, the Chinese flora is also very rich in endemics, with about 12,824 endemic plant species (Huang et al., 2016). However, not less than 10% of higher plants are threatened with extinction in China (Qin et al., 2017). By 2018, a relatively complete biodiversity conservation network has been established in China, including 2,750 nature reserves (NRs; accounting for 14.86% of the land area) and 10 pilot national parks (Gao et al., 2019). Many studies have been conducted to evaluate the effectiveness of these conservation networks for conserving endemic, threatened and national protected species (Jiang et al., 2006; Zhang et al., 2015a; Huang et al., 2016). However, little is known about the effectiveness of *in situ* conservation of seed species in China (Zhang H. N. et al., 2016). Given its geographic diversity, China can serve as a good model for the identification of biodiversity hotspots and conservation priority areas based on massive distribution data and multiple indicators of plant species using comprehensive approaches.

In this study, we attempt to achieve the following goals: (1) to identify biodiversity hotspots for conservation priorities; (2) to evaluate the conservation effectiveness of the current NR networks for seed plants and identify conservation gaps; (3) to put forward conservation strategies and countermeasures for conserving the diversity of seed plants in China. In order to achieve these goals, we compiled an updated inventory of 34,082 seed plants (including infraspecific taxa) and georeferenced 1,007,196 occurrence records, which covered almost all seed plants in China. On the basis of available formation on species distribution, this study is conducted by considering multiple species diversity indexes, species distinctiveness, and spatial phylogenetics. The findings will provide a framework for the conservation of seed plant diversity in China, and present a number of new pathways to strengthen biodiversity conservation.

## Materials and methods

### Database of species occurrence data

We compiled an updated inventory of the seed plants in China based on the *Catalogue of Life China: 2020 Annual Checklist* (Species 2000 China Node^1^). After excluding cultivated and non-native species but including infraspecific taxa (subspecies, varieties and forma) as separate entries, the inventory contained 34,082 seed plants (among which 875 subspecies, 6,116 varieties and 272 forma), which belonged to 3,143 genera and 269 families (Supplementary Table 1.1). Among these, 15,060 species (1,490 genera and 190 families) are endemic to China, and 3,511 species (1,018 genera and 186 families) are threatened with extinction. In addition, there are 33,721 angiosperms (3,100 genera and 259 families. including 14,908 endemic species and 3,361 threatened species) and 361 gymnosperms (among which 120 varieties), which belonged to 43 genera and 10 families, including 152 endemic species and 150 threatened species.

For the occurrence database, we obtained specimen records from the Chinese Virtual Herbarium (CVH^2^) and geo-referenced these specimen records to county level according to the Chinese gazetteer. We used the county as the basic occurrence unit to get comprehensive occurrence data because it is the most widely used unit in describing distribution in botanical literature (Huang et al., 2016; Chi et al., 2017). We also built a distribution database of seed plants at county level according to numerous flora’s and checklists of different provinces and the country. We then integrated the occurrence data from the two above-mentioned databases and built a county-level seed plant database with 1,007,196 items. For each item of the database it is indicated whether it is an endemic species, endangered species, and angiosperms or gymnosperms. Endemism refers to species restricted to a certain geographical area on Earth (Anderson, 1994), and the endemic species of China were identified using to the *Catalogue of Life China.* Threatened species in this study included VU (Vulnerable), EN (Endangered), or CR (Critically endangered) species (Qin et al., 2017).

### Spatial phylogenetic analysis

Phylogenetic trees are of great importance to identify centrers of diversification and thus can contribute to preserve the evolutionary heritage (Véron et al., 2019). Spatial phylogenetics is considered as an important conservation indicator for identifying biodiversity hotspots and the planning of conservation priorities in our study. The largest updated phylogeny for vascular plants, built with the V. PhyloMaker package (Jin and Qian, 2019) was used as a backbone to generate a species-level phylogeny of seed plants in China. In V. PhyloMaker, 32,496 species (accounting for 95.35% of all species in China) were included, belonging to 2,641 genera (84.03%) and 262 families (97.40%; Supplementary Table 1.2). We selected the function *build.nodes. 1* to extract the information about root and basal nodes of the genera to generate a phylogenetic hypothesis of the user-specified species list. We analyzed the distribution patterns of spatial phylogenetics by calculating phylogenetic diversity (PD) and phylogenetic endemism (PE) using Biodiverse V2.0 (Laffan et al., 2010). Based on this software, weighted endemism (WE) was used to study the difference in species range size (Huang et al., 2013). We standardized the values of PD, PE, and WE in each county by calculating the ratios of the value of each index to the maximum value of the corresponding index, and then used the sum of the ratios in each county to measure the level of spatial phylogenetics.

### Identification of biodiversity hotspots

We used an updated administrative map of China from the website of the Ministry of National Resources of the People’s Republic of China^3^ which includes 2,906 county-level administrative units (referred to as the “county”) to map the distribution patterns of hotspots for seed plants or different plant groups such as angiosperms, gymnosperms, endemics, and threatened species. In addition to spatial phylogenetics, we also employed the species richness algorithm and complementary algorithm to better understand the distribution patterns of hotspots. The species richness algorithm defines the regions with the highest species richness in a study area as hotspots (Grenyer et al., 2006; Tang et al., 2006). The complementary algorithm selects the minimum areas that could cover all the species (Dobson et al., 1997; Zhang et al., 2015b; Chi et al., 2017). The complementary algorithm first selected the county with the highest species richness; all species that occurred in this county was excluded from the database while the algorithm searched for the county with the highest number of the remaining species. Once a county with a high conservation value was identified, other counties were selected to complement the previous county, avoiding duplication of priority features (Shrestha and Wang, 2018). This process was continued iteratively until all species were included in the selected counties (Dobson et al., 1997). At the end of the selection, multiple counties that contained the same number of species were selected to avoid missing out on important species.

The hotspots were identified as follows. First, we calculated the distribution patterns of seed plants, endemics, and threatened species using the species richness algorithm and complementary algorithm, and the distribution patterns of spatial phylogenetics, and gained a value (species richness) in each county of corresponding plant taxa by applying the former two algorithms as well as values of PD, PE, and WE from spatial phylogenetics. Second, we standardized the species richness value in each county by calculating the ratio of the value in the county to the county with the highest value under the same distribution pattern. Third, we selected the top 17% counties with higher standardized values as hotspots for different distribution patterns, respectively. Fourth, we summed different standardized values in each county and sorted the sum in descending order. Then, based on previous studies and Aichi Targets (Convention on Biological Diversity, 2010; Chi et al., 2017; Xu et al., 2017), we selected the top 5, 10, and 17% counties with the highest sum in the list of counties first identified as hotspots by more than two algorithms (Supplementary Tables 1.3–1.5). Finally, we assessed the conservation efficiency of the hotspots selected by the top 5, 10, and 17% county areas, and proposed the most appropriate conservation network design to maximize conservation of plant diversity.

### Conservation effectiveness and gap analysis

*In situ* conservation has been considered as the most effective protection means against biodiversity loss (Balmford et al., 1996; IUCN, 2021). So far, there are 2,750 nature reserves (NRs) for biodiversity conservation in China, including 464 national nature reserves (NNRs), 806 provincial nature reserves (PNRs), and many prefectural and county-level NRs. For conservation effectiveness and gap analysis, we only considered NNRs and PNRs because most of prefectural and county-level NRs are not well-managed and their boundaries are not well-defined yet (Quan et al., 2009; Zhang et al., 2015a). In order to evaluate the conservation effectiveness of the current conservation networks and identify conservation gaps, we constructed geo-data files of NNRs and PNRs in ArcGIS based on relevant documents issued by the Ministry of Ecology and Environment of the People’s Republic of China^4^ and documents downloaded from the World Database on Protected Areas^5^. Based on the geographic distributions of the current conservation networks and distribution patterns of hotspots, we measured the conservation effectiveness and identified the gaps by comparing one geodata layer with another, and vice versa. We considered non-overlapping hotspot counties as conservation gaps (Hou et al., 2010; Chi et al., 2017). We calculated the number of species or the proportion of hotspot counties that were overlapping or non-overlapping as a key index for analyzing conservation effectiveness or gaps. Meanwhile, we also assessed the conservation efficiency of the current conservation networks for different plant groups, such as angiosperms, gymnosperms, endemics, and threatened species at country level.

### Analysis on distribution pattern correlation and species composition

We analyzed the correlation of distribution patterns among seed plant species, angiosperms, gymnosperms, endemic species, and threatened species, which were generated based on the species richness algorithm and complementary algorithm in addition to spatial phylogenetics (PD, PE, and WE; Supplementary Table 1.6) in the “corrplot” (version 0.84) package (Wei and Simko, 2017). For the correlation analysis of spatial distribution patterns, we calculated the Pearson coefficient after standardizing relevant variables, which could measure the correlation degree of different spatial distribution patterns in terms of the |r| value (≥0.8 for very strong correlation, 0.6 ≤ |r| <0.8 for strong correlation, 0.4 ≤ |r| <0.6 for moderate correlation, 0.2 ≤ |r| <0.4 for weak correlation, and 0.0 ≤ |r| <0.2 for very weak correlation or no correlation) (Jain and Chetty, 2019). We filled the value of zero (value 0) to the counties without occurrence of species to make the program run smoothly during correlation analysis.

In order to elucidate species composition and internal relationship of different taxonomic groups in protected or unprotected hotspots, as well as conservation effectiveness and gaps of NNRs, PNRs or both, using the “circlize” package (Gu et al., 2014) and “tidyverse” package (Wickham et al., 2019) in software R (version 4.0.2). We presented the species composition of hotspot counties and different plant taxa using chord diagrams and circular barplots. To avoid the overlapping of different plant taxa, threatened species, endemic species (excluding threatened species), and the remaining species (excluding threatened and endemic species) were treated separately based on their conservation priority.

## Results

### Distribution patterns of different plant taxa

**FIGURE 1:**
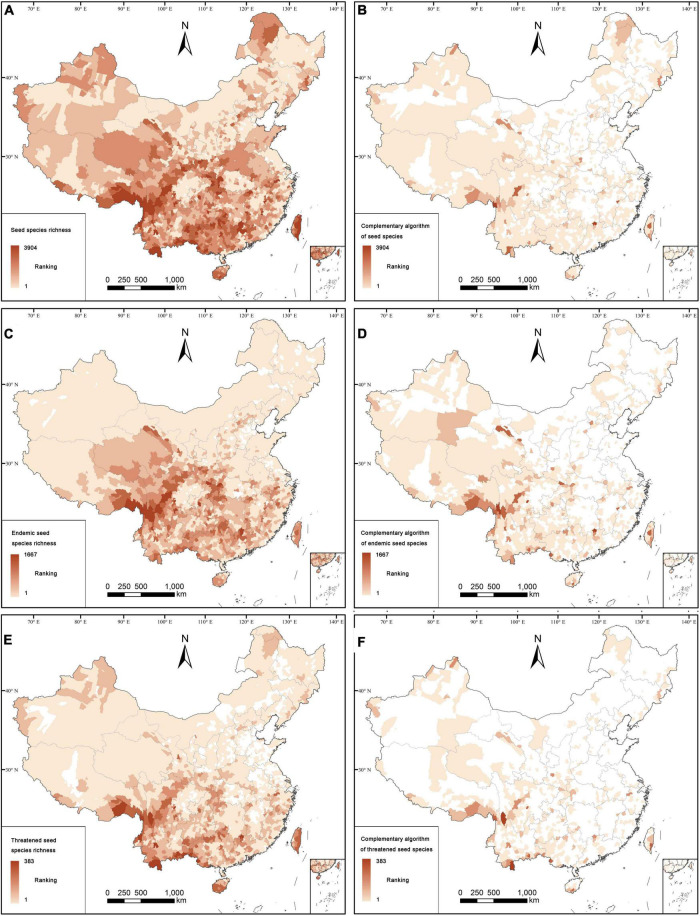
Distribution patterns of all species **(A,B)**, endemics **(C,D)**, and threatened species **(E,F)**. Counties based on all species are determined by **(A)** the species richness algorithm, **(B)** the complementary algorithm; Counties based on endemic species are determined by **(C)** the species richness algorithm, **(D)** the complementary algorithm; Counties based on threatened species are determined by **(E)** the species richness algorithm, **(F)** the complementary algorithm.

For 96.04% of all counties in China, or 2,791 counties, we had species occurrence records; the remaining 3.96% counties with no distribution information on seed plants are mostly located in economically developed regions in eastern China. Three provinces with the highest number of species were Yunnan, Sichuan, and Guangxi (Supplementary Figure 2). According to the species richness algorithm, the distribution patterns of seed plants, angiosperms, gymnosperms, endemics, and threatened species are located in the south-western part of China, especially in south-eastern Xizang, southern Yunnan, western Guangxi, Nanling Mountains, Hengduan Mountains, western Hubei, and eastern Chongqing (Figure 1; Supplementary Figures 3–5). Most endemic species are confined to south-eastern Xizang, Hengduan Mountains, western Hubei, and eastern Chongqing, whereas threatened species are mostly distributed in south-eastern Xizang, southern Hengduan Mountains, and the boundary area of Yunnan.

**FIGURE 2:**
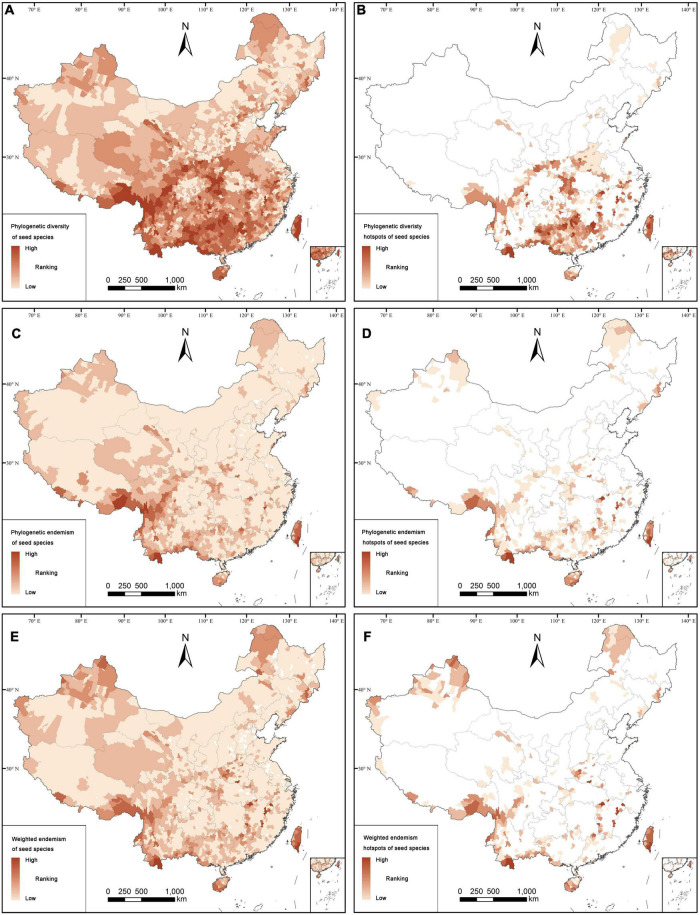
Distribution patterns of phylogenetic diversity [PD **(A,B)**], phylogenetic endemism [PE **(C,D)**], and weight endemism [WE **(E,F)**]. The top 17% of land area with highest values of PD **(B)**, PE **(D)**, and WE **(F)** for seed plant species.

According to the complementary algorithm, there are 1,026 counties (or 35.31% of the Chinese counties) with occurrence records for seed plants. Distribution patterns of seed plants, angiosperms, endemics, and threatened species are concentrated in the western, south-western and southern parts of China, and only few of them are distributed in some areas in the eastern and north-eastern parts of China (Figure 1; Supplementary Figures 3, 4). However, for gymnosperms, including endemics and threatened ones, the counties with high species richness are more distributed in the south-western, central, northern and north-eastern parts of China (Supplementary Figure 5). The analysis of spatial phylogenetics showed that there are 2,691 counties (92.60% of all counties) with high values of PD, PE, and WE for seed species, which are mainly distributed in the south-western and southern parts of China (Figure 2). However, the distribution patterns of PE and WE are more scattered than those of PD. The distribution pattern of PD was mainly found in the southern part of China, and the distribution patterns of PE and WE are found in the south-western, southern, north-western, and north-eastern parts of China.

**FIGURE 3:**
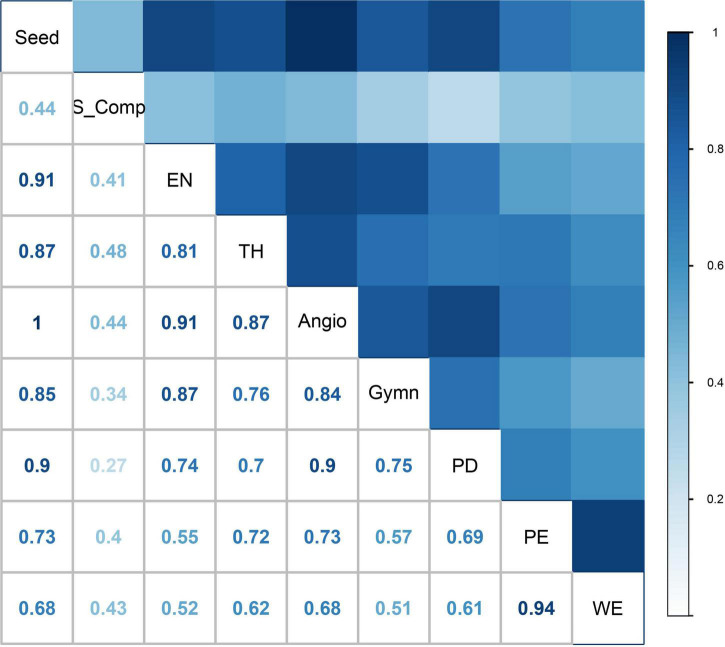
Correlation between species distribution patterns and spatial phylogenetics: all the seed plant species (Seed), angiosperms (Angio), gymnosperms (Gymn), endemic species (EN), and threatened species (TH) according to the species richness algorithm; all the species (S_Comp) according to the complementary algorithm; phylogenetic diversity (PD), phylogenetic endemism (PE), and weighted endemism (WE) according to spatial phylogenetics.

The distribution patterns determined by the species richness algorithm, show a very strong correlation (*r* = 0.81–1.00, *p* < 0.01) among seed plants, angiosperms, gymnosperms, endemic species and threatened species, except for the strong correlation between gymnosperms and threatened species (*r* = 0.76, *p* < 0.01; Figure 3). The distribution patterns determined by the complementary algorithm, show a very strong correlation (*r* = 0.99, *p* < 0.01) between seed plant species and angiosperms, and moderate or weak correlations (*r* = 0.22-0.55, *p* < 0.01) among seed plant species (or angiosperms), gymnosperms, endemics, and threatened species, except for the strong correlation (*r* = 0.64, *p* < 0.01) between seed plants and endemic species (Supplementary Figure 6). In addition, correlation analysis on spatial phylogenetics indicated that there is a strong correlation (*r* = 0.61–0.69, *p* < 0.01) between distribution patterns of PD and PE (Figure 3; Supplementary Figure 6). As for the correlations among distribution patterns of different plant taxa determined by the two algorithms and distribution patterns of spatial phylogenetics, there were moderate or weak correlations (*r* = 0.34–0.48, *p* < 0.01) between the species richness algorithm and complementary algorithm, weak or moderate correlations (*r* = 0.18–0.46, *p* < 0.01) between the complementary algorithm and spatial phylogenetics, and strong or moderate correlations (*r* = 0.51–0.75, *p* < 0.01) between the species richness algorithm and spatial phylogenetics. However, there was an exceptionally strong correlation (*r* = 0.9, *p* < 0.01) between distribution patterns of PD and seed plant species (or angiosperms) determined by the species richness algorithm (Figure 3).

### Distribution patterns of hotspots identified by three algorithms

**FIGURE 4:**
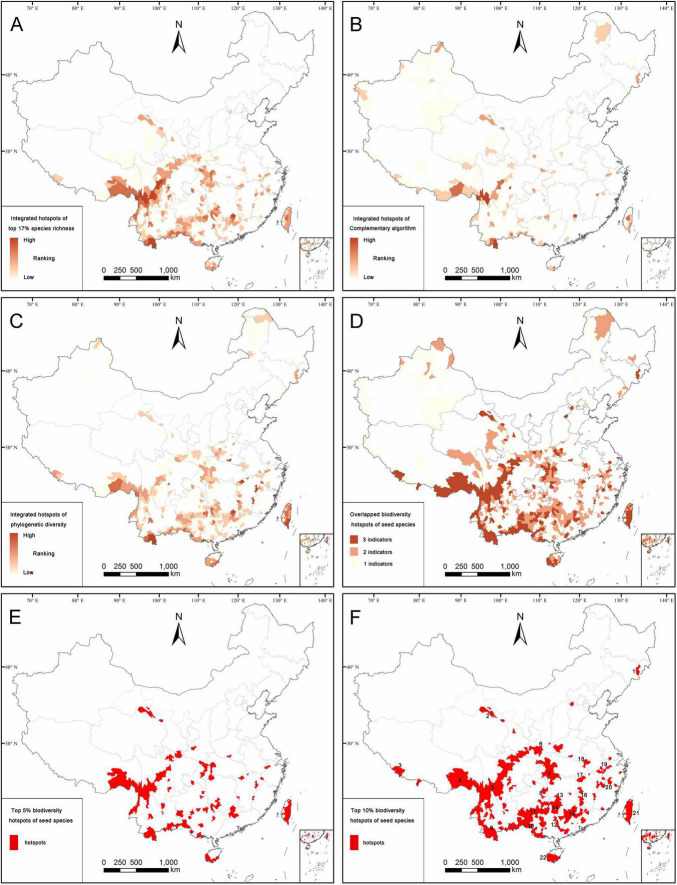
Distribution patterns of the integrated hotspot counties according to the species richness algorithm **(A)**, complementary algorithm **(B)**, spatial phylogenetics **(C)**, the overlapping hotspot counties identified by two or three algorithms **(D)**, top 5% **(E)**, and top 10% **(F)** biodiversity hotpots. The top 10% biodiversity hotspots **(F)** are defined as final biodiversity hotspots including 22 hotspot areas, viz. (1) Northern part of Changbai Mountain, (2) Qilian Mountain, (3) Nielamu region, (4) south-eastern part of Xizang, (5) Hengduan Mountains, (6) Qinling Mountains, (7) Daba-Wushan Mountains, (8) Wuling Mountain, (9) the border areas of Yunnan, (10) the border areas of Guangxi, (11) the boundary areas of Guizhou and Guangxi, (12) Dayao Mountain, (13) southern part of Xuefeng Mountain, (14) western part of Nanling Mountain, (15) eastern part of Nanling Mountain, (16) Luoxiao Mountain, (17) Mufu-Lianyun- Jiuling Mountains, (18) Dabie Mountain, (19) Huangshan-Tianmu Mountains, (20) Wuyi Moutain, (21) Taiwan Island, (22) southern part of Hainan Island.

According to the species richness algorithm, the overlapping hotspot counties of total seed plants, endemics and threatened seed plants are mostly distributed in eastern Qinghai-Tibetan Plateau, south-western and southern China, Hilly Region of Southeast China, western Hubei, and eastern Chongqing (Figure 4A). The hotspots identified by the species richness algorithm covered 432 counties (17.01% of land area), which contained 30,282 seed plants (88.85% of seed plants). There are also 14,016 endemic species (93.07%), 3274 threatened species (93.25%), 29,967 angiosperms (88.87%), and 315 gymnosperms (87.26%) confined to the hotspot counties (Supplementary Table 2.1).

Compared with the species richness algorithm, the overlapping hotspot counties of all seed plants, endemics, and threatened seed plants identified by the complementary algorithm were mainly distributed in the eastern and southern parts of Qinghai-Tibetan Plateau, the south-western, southern and north-western parts of China, and Taiwan (Figure 4B). The hotspots identified by the complementary algorithm covered 240 counties (16.90% of the land area), which contained 31,764 seed plants (93.20% of all seed plants recorded in this study). There were 13,854 endemic species (91.99%), 3309 threatened species (94.25%), 31,420 angiosperms (93.18%), and 344 gymnosperms (95.29%) confined to the hotspot counties identified by the complementary algorithm (Supplementary Table 2.1).

The hotspot counties of spatial phylogenetics identified by phylogenetic diversity (PD), phylogenetic endemism (PE), and weighted endemism (WE) are mostly distributed in the south-eastern part of Xizang, the south-western, southern and north-eastern parts of China, Hilly Region of Southeast China, western Hubei, and eastern Chongqing (Figure 4C), which contained 465 hotspot counties, accounting for 17.04% of land area. Meanwhile, these counties almost covered the main distribution areas of PD, PE, and WE (Figure 2). The hotspot counties identified by the spatial phylogenetics contained 31,502 seed plants, accounting for 92.43% of all seed plants recorded in this study. There are 13,759 endemic species (91.36%), 3295 threatened species (93.85%), 31,155 angiosperms (92.39%), and 347 gymnosperms (96.12%; Supplementary Table 2.1).

### Distribution patterns of biodiversity hotspots

After the overlapping of the top 17% hotspot counties, identified by the species richness algorithm, complementary algorithm and spatial phylogenetics, respectively, 579 counties are identified as overlapping hotspot counties, accounting for 27.13% of land area of China (Figure 4D). Among these, 397 counties were jointly identified by two or three algorithms, and they were regarded as biodiversity hotspot counties of seed plants, which accounted for 16.37% of the total area of China (close to the goals of 17% of global protected land in Aichi Target 11). These biodiversity hotspots contained 31,637 seed plant species (92.83% of seed plant species) belonging to 3,044 genera and 265 families, 14,020 endemic species (93.09%), 3,311 threatened species (94.30%), 31,296 angiosperms (92.81%), and 341 gymnosperms (94.46%; Supplementary Table 2.2). Furthermore, we identified 5 and 10% of China’s land area with the highest conservation values as the top 5% and top 10% biodiversity hotpots, containing 110 and 264 counties, respectively (Figures 4E,F). The top 5% biodiversity hotspots contained 26,189 seed plant species (76.84%), 12,080 endemic species (80.21%), and 2,868 threatened species (81.69%; Supplementary Table 2.2). The top 10% biodiversity hotspots harbored 29,045 seed plant species (85.22%), 13,248 endemic species (87.97%), and 3,164 threatened species (90.12%; Supplementary Table 2.2). Given the limited resource investment and threatened species as the most important plant groups for biodiversity conservation, we took 90% of all threatened species as a threshold for biodiversity priority. Finally, the top 10% biodiversity hotspots were considered the most important hotspots and defined as the ultimate seed plant hotspots for China. For these hotspots, we evaluated their conservation effectiveness and analyzed the conservation gaps.

Based on geographic distribution of the 264 hotspot counties, which represent the top 10% of biodiversity hotspots, 22 biodiversity hotspot areas were determined, including (1) the northern part of Changbai Mountain, (2) Qilian Mountain, (3) Nielamu region, (4) the south-eastern part of Xizang, (5) Hengduan Mountains, (6) Qinling Mountains, (7) Daba-Wushan Mountains, (8) Wuling Mountain, (9) the boundary areas of Yunnan, (10) the boundary areas of Guangxi, (11) the boundary areas of Guizhou and Guangxi, (12) Dayao Mountain, (13) the southern part of Xuefeng Mountain, (14) the western part of Nanling Mountain, (15) the eastern part of Nanling Mountain, (16) Luoxiao Mountain, (17) Mufu-Lianyun- Jiuling Mountains, (18) Dabie Mountain, (19) Huangshan-Tianmu Mountains, (20) Wuyi Mountain, (21) Taiwan Island, (22) the southern part of Hainan Island (Figure 4F; Supplementary Table 2.3). In general, these biodiversity hotspot areas of seed plants are located in the mountainous areas of China, especially in the eastern and south-eastern parts of Xizang, Hengduan Mountains, Yun-Gui Plateau, mountainous areas of South China, Hilly Region of Southeast China, Qinling Mountains, and Daba-Wushan Mountains.

### Conservation effectiveness of the current conservation network

**FIGURE 5:**
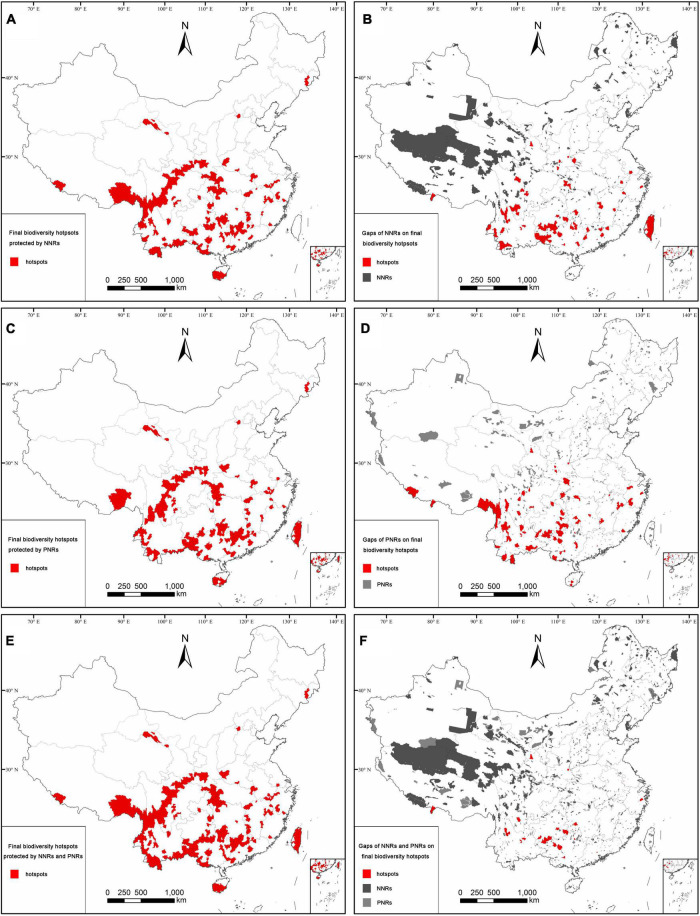
Conservation effectiveness and gaps in the final biodiversity hotspots of seed plant species in the current conservation network: conservation effectiveness of NNRs **(A)**, PNRs **(C)**, and NNRs and PNRs **(E)**; conservation gaps of NNRs **(B)**, PNRs **(D)**, and NNRs and PNRs **(F)**.

Conservation effectiveness analysis indicated that there are 181 out of the 264 final hotspot counties protected by NNRs. These are mainly distributed in south-eastern Xizang, Hengduan Mountains, the boundary areas of Yunnan, the boundary areas of Guangxi, the northern boundary area of Guangxi, the eastern and western parts of Nanling Mountains, southern Hainan Island, Hilly Region of Southeast China, Daba-Wushan Mountains, and Qilian Mountains (Figure 5A). The hotspot counties covered by NNRs harbored 25,912 seed plants (76.03% of all seed plants), 25,645 angiosperms (76.05%), 267 gymnosperms (73.96%), 12,079 endemic species (80.21%), and 2,887 threatened species (82.23%; Supplementary Table 2.4). In addition, there are 177 hotspot counties (or 67.05% of the 264 ultimate hotspot counties) protected by PNRs, which contained 25,848 seed plants (75.84% of all seed plants), 25,572 angiosperms (75.83%), 276 gymnosperms (76.45%), 11,515 endemic species (76.46%), and 2,762 threatened species (78.67%; Supplementary Table 2.4). The hotspot counties protected by PNRs are mostly confined to south-eastern Xizang, Hengduan Mountains, the boundary areas of Yunnan, the southern and south-eastern parts of China, Taiwan Island, Daba-Wushan Mountains, and Qilian Mountains (Figure 5C).

Taken together, 239 out of the 264 hotspot counties were well-protected in the current conservation network of NNRs and PNRs, with high conservation values in each hotspot area of seed plants (Figure 5E), and they harbored 28,584 seed plants (83.87% of all seed plants), 28,286 angiosperms (83.88%), 298 gymnosperms (82.55%), 13,058 endemic species (86.71%), and 3,124 threatened species (88.98%; Supplementary Table 2.4). With regard to their conservation effectiveness, 22 hotspot areas showed better conservation effectiveness for more hotspot counties, such as south-eastern Xizang, northern Hengduan Mountains, the boundary areas of Yunnan, the boundary areas of Guangxi, Dayao Mountain, Nanling Mountain, Mufu-Lianyun- Jiuling Mountains, Luoxiao Mountain, southern Hainan, Taiwan, Wuyi Mountain, Huangshan Mountain, Dabie Mountain, Qinling Mountains, Daba-Wushan Mountains, Wuling Mountain, Qilian Mountains, and the northern part of Changbai Mountain (Figure 5E).

The counties covered by NNRs, PNRs, and NNRs-PNRs accounted for 58.60, 59.34, and 79.22% of the total land area of China, respectively. The counties covered by NNRs contained 29,828 seed plants (87.52% of all seed plants), 29,522 angiosperms (87.55%), 306 gymnosperms (84.76%), 13,448 endemic species (89.30%), and 3,138 threatened species (89.38%). The counties protected by PNRs harbored 30,300 seed plants (88.90% of all seed plants), 29,974 angiosperms (88.89%), 326 gymnosperms (90.30%), 13,097 endemic species (86.97%), and 3,072 threatened species (87.50%). There are 1,560 out of 2,906 counties protected in the current conservation network of NNRs and PNRs, which contained 33,053 seed plants (96.98% of all seed plants), 32,707 angiosperms (96.99%), 346 gymnosperms (95.84%), 14,631 endemic species (97.15%), and 3,406 threatened species (97.01%; Supplementary Table 2.4).

### Conservation gaps of the conservation network

Conservation gap analysis showed that 83 hotspot counties (31.44% of the 264 hotspot counties) are outside NNRs, which contained 19,009 seed plant species (55.77% of all seed plants), 18,784 angiosperms (55.70% of all angiosperms), 225 gymnosperms (62.33% of all gymnosperms), 8031 endemic species (53.33% of all endemic species), and 1,772 threatened species (50.47% of all threatened species; Supplementary Table 2.4). The hotspot counties unprotected by NNRs are mainly distributed in the southern part of Hengduan Mountains, the boundary areas of Southwest Yunnan, the boundary areas of Guizhou and Guangxi, the western part of Guangxi, the boundary areas of Guangxi and Guangdong, and Taiwan Island (Figure 5B).

There are 87 hotspot counties (32.95% of all hotspot counties) outside PNRs, which harbored 20,972 seed plant species (61.53% of all seed plant species), 20,736 angiosperms (61.49% of all angiosperms), 236 gymnosperms (65.37% of all gymnosperms), 9441 endemic species (62.69% of all endemic species), and 2,265 threatened species (64.51% of all threatened species; Supplementary Table 2.4). The hotspot counties uncovered by PNRs are mainly distributed in the Nielamu region, south-eastern Xizang, southern Hengduan Mountains, the boundary areas of South Yunnan, the boundary areas of Guizhou and Guangxi, southern Xuefeng Mountain, and Hilly Region of Southeast China (Figure 5D).

For gaps of NNRs and PNRs, 25 out of the 264 hotspot counties (9.47% of all hotspot counties) are unprotected by the current both conservation networks. These are mainly distributed in the south-eastern part of Hengduan Mountains, eastern Yunnan, southern Guizhou, and north-western Guangxi (Figure 5F). These counties harbor 9,862 seed plants (accounting for 28.94% of all seed plants), 9712 angiosperms (28.80% of all angiosperms), 150 gymnosperms (41.55% of all gymnosperms), 3891 endemic species (25.84% of all endemic species), and 766 threatened species (21.82% of all threatened species; Supplementary Table 2.4).

### Conservation efficiency of hotspots, NNRs, and PNRs for different plant taxa

The analysis of the species composition of seed plants in the hotspots, NNRs, and PNRs indicated that the hotpots only cover 10% of land area of China and contained only 264 counties (this is 9% of all counties) but harbored more seed plant species (29,045 species, including 3,164 threatened species and 11,113 endemic species excluding threatened species). The NNRs cover 58.60% of the land area and 901 counties (31% of all counties), and harbor 29,828 species, including 3,138 threatened species and 11,342 endemic species (excluding threatened species) while the PNRs cover 59.34% of the land area and 1,182 counties (40.7% of all counties), and harbor 30,300 species including 3,072 threatened species and 11,037 endemic species (excluding threatened species; Supplementary Figure 7 and Supplementary Table 2.5). Compared to PNRs, NNRs contained fewer species, but more threatened and endemic species (Supplementary Figure 7). For the conservation effectiveness of the current conservation network, there were 1,560 counties (53.7%) covered by NNRs and PNRs, which contained 33,053 species including 3,406 threatened species and 12,331 endemic species (excluding threatened species) (Supplementary Figure 7).

**FIGURE 6:**
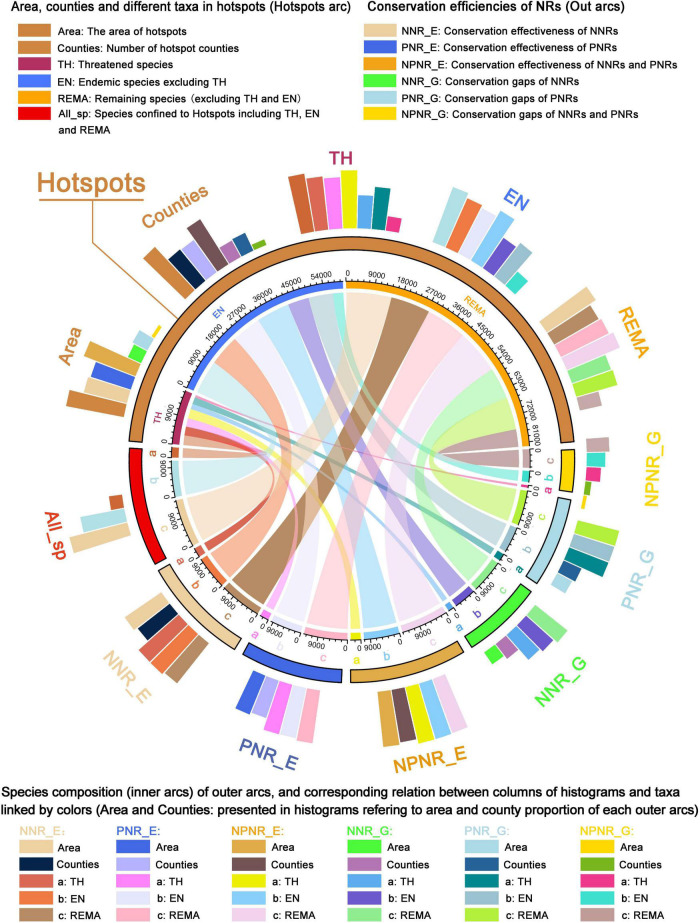
Chord diagram and circular barplot showing the connections between the number and area of hotspot counties and species composition of seed plants in the hotspots, conservation effectiveness, and conservation gaps of NNRs and PNRs. The inner arcs are connected to the circular barplot of the same color to represent the same group. The colored fragments in the inner arc represent the number of species of a certain taxonomic group, or the aggregation of the hotspots, conservation effectiveness and conservation gaps of one taxonomic group. The ratio of the number of species of each taxon to the number of species of all species in hotspots is shown in All_sp.

The NNRs, PNRs and both together, there were 25,912 species (2,887 threatened species and 10,146 endemic species excluding threatened species), 25,848 species (2,762 threatened species and 9,673 endemic species excluding threatened species), and 28,584 species (3,124 threatened species and 10,957 endemic species excluding threatened species), respectively (Figure 6; Supplementary Table 2.5). Although the area and number of hotspot counties decreased, especially in NNRs, there are still a large number of threatened and endemic species in the gaps of the NNRs (1,772 threatened species and 6,907 endemic species excluding threatened species) or the PNRs (2,265 threatened species and 7,996 endemic species excluding threatened species; Figure 6). The gaps in NNRs and PNRs contained 25 hotspot counties (0.51% land area of China) but contained 28.9% of seed plants in China, including 21.8% of all threatened species and 26.98% of all endemic species (excluding threatened species). The relationships between species composition and taxa proportion in each area (hotspots, NNRs, PNRs, effectiveness, and gaps) are presented in more detail in Supplementary Figure 7 and Supplementary Table 2.5.

## Discussion

### Biodiversity hotspots and significance for conservation

In this study, we used for the first time the complementary algorithm and spatial phylogenetics to explore the spatial distribution patterns and biodiversity hotspots of the seed plants in China, in order to identify hotspots with a unique flora (Zhang et al., 2015b; Chi et al., 2017) and conservation priority areas with rich evolutionary history (Chen and Li, 2018). The numbers of all seed plants, angiosperms, gymnosperms, endemics, and threatened species confined to the overlapping hotspot areas identified by the complementary algorithm were higher than those identified by the species richness algorithm and the spatial phylogenetics (Supplementary Table 1.1). In addition, there was a very strong correlation between the phylogenetic diversity (PD) and taxonomic diversity of seed plants (or angiosperms) (Figure 3); a similar result was found in an earlier study for terrestrial vertebrates (Quan et al., 2018). The final hotspots identified in this study not only show high species richness, but are also characterized by rich evolutionary information. The formation of these hotspots may be the result of climate or soil variability, complex terrain and the strong environmental gradients in mountainous areas which promoted the isolation and diversification of seed plant species; the plains were dominated by agriculture, but the mountainous areas with a low degree of human disturbance and a diverse ecological environment (Körner and Spehn, 2002; Tang et al., 2006; Svenning et al., 2010; Huang et al., 2011; Stein et al., 2014).

We identified 22 biodiversity hotspots for seed plants in China, which were concentrated in the mountains of South China. These hotspots cover only 10% of the Chinese land area and 9% of the counties in China, but contained 29,045 seed plant species which is 85.22% of all seed plant species (Supplementary Table 2.4). The total area of the hotspot counties is about 6 times less than that covered by NNRs or PNRs, and the number of hotspot counties was about 3.4 or 4.5 times lower than that protected by NNRs or PNRs. However, the newly defined hotspot counties contained more threatened species (3,164 threatened species) than NNRs (3,138 threatened species) or PNRs (3,072 threatened species; Supplementary Figure 7 and Supplementary Table 2.5). The biodiversity hotspots identified in this study deserve much attention because of to their most abundant plant resources, which are mainly distributed in the eastern part of Qinghai-Tibet plateau, Hengduan Mountains, Yun-Gui Plateau, Daba-Wushan Mountains, Hilly Region of Southeast China, and Qinling Mountains (Figure 4F). These areas provided habitats for three types of vegetation, including subtropical evergreen broad-leaved forest, tropical seasonal forest, and tropical rainforest (Wu, 1980). Since the conservation value of mountains for seed plant diversity in China had already been recognized (Tang et al., 2006; Wang et al., 2011; Huang et al., 2012; Zhao et al., 2016), it is urgent to uncover the spatial distribution patterns and identify conservation priority areas for seed plants more accurately based on the comprehensive distribution information and systematic methods.

Our study has confirmed almost all hotspot areas identified in previous studies based on limited distribution information, a specific taxon or a single indicator (Tang et al., 2006; Zhang and Ma, 2008; Huang et al., 2016; Zhao et al., 2016; Chi et al., 2017; Xu et al., 2017). Moreover, by using the complementary algorithm and spatial phylogenetics, we identified some new hotspots with high conservation value that were ignored in previous studies, such as Dabie Mountain, Mufu-Lianyun-Jiuling Mountains, northern Qilian Mountain, and northern Changbai Mountain (Figure 4F). In addition, we identified some hotspot areas that were rarely identified previously, such as Nielamu region and Luoxiao Mountain. Although 32 biodiversity conservation priority areas in China had been defined before (Liu et al., 2017), some areas with high species richness and important evolutionary history had not been considered yet as priority areas for biodiversity conservation, such as western Yunnan, Wushan Mountain, and eastern Guangxi. Nevertheless, there are still some limitations due to the distribution information of species at county level in this study. For example, we only considered the biodiversity hotspot counties protected by the current conservation networks of NNRs and PNRs. However, when the NR or only part of a reserve was distributed in the hotspot county, the conservation effectiveness of the NNRs could be inevitably overestimated while the urgency of conservation for seed plants was underestimated.

### Optimization the conservation network and suggestions for conservation

Although NNRs were generally regarded as the best among all types of protection areas in terms of conservation effectiveness (Wu et al., 2011), there were still 83 hotspot counties uncovered by NNRs, which are mainly situated in the south-western and southern parts of China, containing 50.47% of threatened species and 54.48% of endemic species (excluding threatened species) in China (Figures 5B, 6; Supplementary Table 2.5). Therefore, new NNRs, micro-reserves, and ecological corridors, etc., should be established in the future to fill the conservation gaps in the existing NNRs. The PNRs played an important complementary role in biodiversity conservation, especially in south-western Yunnan, southern Sichuan, the boundary areas of Guangxi and Guangdong, and Taiwan (Figures 5C,D). There were 58 out of 264 hotspot counties exclusively protected by PNRs (Figures 5C, 6). Interestingly, the gaps in PNRs were also confined to southwest China, suggesting that the hotspots clustered in southwest China received insufficient conservation. Taken together, a total of 25 hotspot counties confined to southwest China were uncovered by either NNRs or PNRs. These only accounted for 0.51% of the total land area but contained 21.82% of all threatened species and 26.98% of all endemic species (excluding threatened species) in China (Figure 6; Supplementary Table 2.5). Therefore, it is necessary to strengthen conservation efforts by establishing new NNRs and PNRs in order to improve the conservation effectiveness of the current conservation networks.

In addition to creating NRs, some national parks and ecological corridors should be established in the provincial boundary areas (Považan et al., 2014; Zheng et al., 2019). For example, the conservation status of hotspot counties distributed in the boundary areas of Southwest Yunnan, the boundary areas of Yunnan, Guizhou, and Guangxi, and the boundary areas of Guizhou, Guangxi, and Hunan has been severely affected by the provincial administrative divisions (Figures 4F, 5). Therefore, we advise to create nature reserves that cross the borders of the provincial administrative boundaries (Xu et al., 2017; Su et al., 2019). In addition, establishing a conservation network of integrated geographical areas unimpeded by the cross-border provincial administrative divisions, can reduce human disturbance and habitat fragmentation, enhance gene flow between communities, and protect biodiversity more comprehensively. Recently, China is speeding up the establishment of a NR system dominated by national parks (Zhou and Grumbine, 2011; Zhang L. et al., 2017), and we believe that systematic research on biodiversity can improve the current biodiversity conservation.

### Strengthening the targeted conservation on seed plants and specific taxa

Previous studies indicate that there are clear mismatches in the distribution patterns of hotspots between different plant taxa (Tang et al., 2006; Zhang et al., 2021). However, limited conservation efforts have been made to implement targeted conservation actions focusing on specific taxa (Yang et al., 2021). The biodiversity hotspots identified in this study, covering 10% of the land area and containing 85.22% of seed plant species, are representative for a series of taxa and multiple conservation indicators. However, based on the correlation of the distribution patterns between specific plant groups and different algorithms, we need to protect not only hotspot areas but also key plant taxa. Given the vast territory of China and its highly diversified flora, there was a weak or moderate correlation among the distribution patterns of seed plant species (or angiosperms), gymnosperms, endemics and threatened species according to the complementary algorithm, although there was a very strong correlation between them according to the species richness algorithm (except for a strong correlation between gymnosperms and threatened species; Figure 3).

The distribution patterns of seed plant species (or angiosperms), gymnosperms, endemics, and threatened species were different, and therefore, specific conservation efforts should be applied focusing on different plant taxa. For example, species richness of endemic species was relatively concentrated in south-eastern Xizang, Hengduan Mountains, western Hubei, and eastern Chongqing compared to that of seed plants and threatened species (Figure 1C; Supplementary Figure 3). Species richness of threatened species was more concentrated in the boundary areas of South Yunnan, the boundary areas of Guangxi, and south-eastern Xizang (Figure 1E; Supplementary Figure 3), possibly due to the presence of low disturbance intensity, proper climate and high habitat heterogeneity in these areas (Wang et al., 2011; Zhang et al., 2015a) and this pattern deserves further studies. Although the gymnosperms were mostly concentrated to the counties with high species richness of seed plants, they were also scattered distributed in other areas, such as south-eastern Qinghai, south-western Yunnan, Alxa Plateau, and northern Xinjiang (Supplementary Figure 5). In addition, the distribution patterns of different plant groups identified by the complementary algorithm were markedly different from those by the species richness algorithm, and the distribution of seed plant species identified by the complementary algorithm was concentrated in the western and south-western regions of China, which was of great significance for the identification of the special flora of China (Figure 1; Supplementary Figures 3–5).

## Conclusion

The combination of three methods, namely the species richness algorithm, complementary algorithm, and spatial phylogenetics, led to the identification of priority areas for seed plant conservation in China more accurate and scientific than before which will subsequently lead to new guidelines for long-term biodiversity conservation. We may conclude that the conservation of the hotspot areas identified in this study could maximize biodiversity conservation in China with minimum land area and minimal damage to existing biodiversity resources for economic development. It is important to increase the conservation effectiveness of the current conservation networks by systematically and comprehensively identifying biodiversity hotspots and putting forward an integrative conservation priority planning framework. This study identified biodiversity hotspots and conservation priority areas for seed plants in China based on over a million geographical distributed data and multiple conservation indicators, such as species richness, complementarity and spatial phylogenetics. Furthermore, we analyzed the conservation effectiveness and gaps in the current conservation networks and put forward some targeted and feasible suggestions and countermeasures for biodiversity conservation.

## Data availability statement

The original contributions presented in this study are included in the article/Supplementary material, further inquiries can be directed to the corresponding authors.

## Author contributions

XY performed material preparation and investigation. XY, WZ, and FQ performed methodology and software and wrote the draft of the manuscript. SY, ES, and JW performed material preparation, methodology, manuscript revision, and supervision. All authors contributed to the study conception and design, commented on previous versions, reviewed the manuscript, read, and approved the final manuscript.

## Footnotes

1
http://www.sp2000.org.cn

2
http://www.cvh.ac.cn/

3
http://bzdt.ch.mnr.gov.cn/

4
http://www.mee.gov.cn

5
https://www.protectedplanet.net/

## References

[B1] Anderson S. (1994). Area and Endemism. *Q. Rev. Biol.* 69 451–471.

[B2] Balmford A. Mace G. M. Leader-Williams N. (1996). Designing the ark: Setting priorities for captive breeding. *Conserv. Biol.* 10 719–727.

[B3] Brooks T. M. Mittermeier R. A. da Fonseca G. A. B. Gerlach J. Hoffmann M. Lamoreux J. F. (2006). Global Biodiversity Conservation Priorities. *Science* 313 58–61. 16825561 10.1126/science.1127609

[B4] Brum F. T. Graham C. H. Costa G. C. Hedges S. B. Penone C. Radeloff V. C. (2017). Global priorities for conservation across multiple dimensions of mammalian diversity. *Proc. Natl. Acad. Sci. U.S.A.* 114 7641–7646. 10.1073/pnas.1706461114 28674013 PMC5530698

[B5] Chen Z. Li D. (2018). Phylogenetic diversity and biodiversity conservation. *Science* 70 22–25. 10.3969/j.issn.0368-6396.2018.05.008

[B6] Chi X. Zhang Z. Xu X. Zhang X. Zhao Z. Liu Y. (2017). Threatened medicinal plants in China: Distributions and conservation priorities. *Biol. Conserv.* 210 89–95. 10.1016/j.biocon.2017.04.015

[B7] Convention on Biological Diversity (2010). *Strategic Plan for Biodiversity 2011-2020.* Available online at: https://www.cbd.int/decision/cop/?id=12268 (accessed January 15, 2017).

[B8] Dobson A. P. Rodriguez J. P. Roberts W. M. Wilcove D. S. (1997). Geographic Distribution of Endangered Species in the United States. *Science* 275 550–553. 8999803 10.1126/science.275.5299.550

[B9] Faith D. P. (2013). Biodiversity and evolutionary history: Useful extensions of the PD phylogenetic diversity assessment framework. *Ann. N. Y. Acad. Sci.* 1289 69–89. 10.1111/nyas.12186 23773093

[B10] Gao J. X. Xu M. J. Zou C. X. (2019). Development Achievement of Natural Conservation in 70 Years of New China. *Environ. conformity Assess.* 11 25–29. 10.16868/j.cnki.1674-6252.2019.04.025

[B11] Grenyer R. Orme C. D. Jackson S. F. Thomas G. H. Davies R. G. Davies T. J. (2006). Global distribution and conservation of rare and threatened vertebrates. *Nature* 444 93–96. 10.1038/nature05237 17080090

[B12] Gu Z. Gu L. Eils R. Schlesner M. Brors B. (2014). circlize Implements and enhances circular visualization in R. *Bioinformatics* 30 2811–2812. 10.1093/bioinformatics/btu393 24930139

[B13] Hou M. F. Jordi L. P. Qin H. N. Wang L. S. Liu Y. (2010). Distribution pattern and conservation priorities for vascular plants in southern China: Guangxi province as a case study. *Bot. Stud.* 51 377–386.

[B14] Huang J. Chen B. Liu C. Lai J. Zhang J. Ma K. (2012). Identifying hotspots of endemic woody seed plant diversity in China. *Divers. Distrib.* 18 673–688. 10.1111/j.1472-4642.2011.00845.x

[B15] Huang J. Chen J. Ying J. Ma K. (2011). Features and distribution patterns of Chinese endemic seed plant species. *J. Syst. Evol.* 49 81–94. 10.1111/j.1759-6831.2011.00119.x

[B16] Huang J. Huang J. Liu C. Zhang J. Lu X. Ma K. (2016). Diversity hotspots and conservation gaps for the Chinese endemic seed flora. *Biol. Conserv.* 198 104–112. 10.1016/j.biocon.2016.04.007

[B17] Huang J. Zhang J. Yang Y. Ma K. (2013). Advances in methods for measuring patterns of endemic plant diversity. *Biodivers. Sci.* 21 99–110. 10.3724/sp.J.1003.2013.12175

[B18] IUCN (2021). *IUCN red Iist of threatened species. Version 2021–3.* IUCN, Switzerland. Available online at: https://www.iucnredlist.org (accessed July 7, 2021).

[B19] Jain R. Chetty P. (2019). *How to interpret results from the correlation test?.* Available online at: https://www.projectguru.in/how-to-interpret-results-from-the-correlation-test/

[B20] Jiang M. K. Wang Z. Qin W. H. He Z. H. (2006). Effectiveness of National Priority Wildlife Protection in Nature Reserves. *J. Ecol. Rural Environ.* 22 35–38.

[B21] Jin Y. Qian H. (2019). V.PhyloMaker: An R package that can generate very large phylogenies for vascular plants. *Ecography* 42 1353–1359. 10.1111/ecog.04434 PMC9363651 35967255

[B22] Kelly S. Grenyer R. Scotland R. W. Austin J. (2014). Phylogenetic trees do not reliably predict feature diversity. *Divers. Distrib.* 20 600–612. 10.1111/ddi.12188

[B23] Körner C. Spehn E. M. (2002). *Mountain Biodiversity: A Global Assessment.* New York: The Parthenon Publishing Group.

[B24] Kougioumoutzis K. Kokkoris I. P. Panitsa M. Trigas P. Strid A. Dimopoulos P. (2020). Spatial Phylogenetics, Biogeographical Patterns and Conservation Implications of the Endemic Flora of Crete (Aegean, Greece) under Climate Change Scenarios. *Biology* 9:199. 10.3390/biology9080199 32751787 PMC7463760

[B25] Laffan S. W. Lubarsky E. Rosauer D. F. (2010). Biodiverse, a tool for the spatial analysis of biological and related diversity. *Echography* 33 643–647. 10.HH/j.1600-0587.2010.06237.x

[B26] Le Bagousse-Pinguet Y. Soliveres S. Gross N. Torices R. Berdugo M. Maestre F. T. (2019). Phylogenetic, functional, and taxonomic richness have both positive and negative effects on ecosystem multifunctionality. *Proc. Natl. Acad. Sci. U.S.A.* 116 8419–8424. 10.1073/pnas.1815727116 30948639 PMC6486734

[B27] Liang Q. Xu X. Mao K. Wang M. Wang K. Xi Z. (2018). Shifts in plant distributions in response to climate warming in a biodiversity hotspot, the Hengduan Mountains. *J. Biogeogr.* 45 1334–1344. 10.1111/jbi.13229

[B28] Liu H. M. Gao J. X. Zhang H. Y. Ma X. L. Xu X. L. (2017). Human disturbance monitoring and assessment in the biodiversity conservation priority area China. *J. Geo-Inf. Sci.* 19 1456–1465. 10.1073/pnas.2113416118 34983877 PMC8764696

[B29] Luo Z. Tang S. Jiang Z. Chen J. Fang H. Li C. (2016). Conservation of Terrestrial Vertebrates in a Global Hotspot of Karst Area in Southwestern China. *Sci. Rep.* 6:25717. 10.1038/srep25717 27228463 PMC4881395

[B30] Marchese C. (2015). Biodiversity hotspots: A shortcut for a more complicated concept. *Glob. Ecol. Conserv.* 3 297–309. 10.1016/j.gecco.2014.12.008

[B31] Monastersky R. (2014). Biodiversity: Life - a status report. *Nature* 516 159–161. 10.1038/516158a 25503217

[B32] Myers N. Mittermeier R. A. Mittermeier C. G. Da Fonseca G. A. B. Kent J. (2000). Biodiversity hotspots for conservation priorities. *Nature* 403 853–858. 10706275 10.1038/35002501

[B33] Orme C. D. Davies R. G. Burgess M. Eigenbrod F. Pickup N. Olson V. A. (2005). Global hotspots of species richness are not congruent with endemism or threat. *Nature* 436 1016–1019. 10.1038/nature03850 16107848

[B34] Pimm S. L. Jenkins C. N. Abell R. Brooks T. M. Gittleman J. L. Joppa L. N. (2014). The biodiversity of species and their rates of extinction, distribution, and protection. *Science* 344:1246752. 10.1126/science.1246752 24876501

[B35] Pitman N. C. Jørgensen P. M. (2002). Estimating the size of the world’s threatened flora. *Science* 298:989. 10.1126/science.298.5595.989 12411696

[B36] Považan R. Getzner M. Švajda J. (2014). Value of Ecosystem Services in Mountain National Parks. Case Study of Vel’ká FatraNational Park (Slovakia). *Pol. J. Environ. Stud.* 23 1699–1710.

[B37] Qin H. Yang Y. Dong S. He Q. Jia Y. Zhao L. (2017). Threatened Species List of China’s Higher Plants. *Biodivers. Sci.* 25 696–744. 10.17520/biods.2017144 34063014

[B38] Quan J. Ouyang Z. Y. Xu W. H. Miao H. (2009). Management effectiveness of China nature reserves status quo assessment and counter measures. *Chinese J. Appl. Ecol.* 20 1739–1746. 10.13287/j.1001 19899479

[B39] Quan Q. Che X. Wu Y. Wu Y. Zhang Q. Zhang M. (2018). Effectiveness of protected areas for vertebrates based on taxonomic and phylogenetic diversity. *Conserv. Biol.* 32 355–365. 10.1111/cobi.12986 28703325

[B40] Ren H. Duan Z. Y. (2017). *The Theory and Practice on Construction of Classic Botanical Garden.* Beijing: Science Press.

[B41] Shrestha N. Wang Z. (2018). Selecting priority areas for systematic conservation of Chinese Rhododendron: Hotspot versus complementarity approaches. *Biodivers. Conserv.* 27 3759–3775. 10.1007/s10531-018-1625-8

[B42] Stein A. Gerstner K. Kreft H. (2014). Environmental heterogeneity as a universal driver of species richness across taxa, biomes and spatial scales. *Ecol. Lett.* 17 866–880. 10.1111/ele.12277 24751205

[B43] Su X. Han W. Liu G. Zhang Y. Lu H. (2019). Substantial gaps between the protection of biodiversity hotspots in alpine grasslands and the effectiveness of protected areas on the Qinghai-Tibetan Plateau. *China. Agric. Ecosyst. Environ.* 278 15–23. 10.1016/j.agee.2019.03.013

[B44] Svenning J.-C. Fitzpatrick M. C. Normand S. Graham C. H. Pearman P. B. Iverson L. R. (2010). Geography, topography, and history affect realized-to-potential tree species richness patterns in Europe. *Ecography* 33 1070–1080. 10.1111/j.1600-0587.2010.06301.x

[B45] Tang Z. Wang Z. Zheng C. Fang J. (2006). Biodiversity in China’s mountains. *Front. Ecol. Environ.* 4:347–352. 10.1890/1540-92952006004

[B46] Teller B. J. Miller A. D. Shea K. Cadotte M. (2015). Conservation of passively dispersed organisms in the context of habitat degradation and destruction. *J. Appl. Ecol.* 52 514–521. 10.1111/1365-2664.12379

[B47] Venter O. Fuller R. A. Segan D. B. Carwardine J. Brooks T. Butchart S. H. (2014). Targeting global protected area expansion for imperiled biodiversity. *PLoS Biol.* 12:e1001891. 10.1371/journal.pbio.1001891 24960185 PMC4068989

[B48] Véron S. Saito V. Padilla-Garcia N. Forest F. Bertheau Y. (2019). The Use of Phylogenetic Diversity in Conservation Biology and Community Ecology A Common Base but Different Approaches. *Q. Rev. Biol.* 94 123–148.

[B49] Volis S. (2018). Securing a future for China’s plant biodiversity through an integrated conservation approach. *Plant Divers.* 40 91–105. 10.1016/j.pld.2018.04.002 30175290 PMC6114126

[B50] Wang W. Li J. (2021). In-situ conservation of biodiversity in China: Advances and prospects. *Biodivers. Sci.* 29 133–149. 10.17520/biods.2020070 34063014

[B51] Wang Z. Fang J. Tang Z. Lin X. (2011). Patterns, determinants and models of woody plant diversity in China. *Proc. Royal Soc. B.* 278 2122–2132. 10.1098/rspb.2010.1897 21147804 PMC3107620

[B52] Wei T. Y. Simko V. (2017). *R package “corrplot”: Visualization of a Correlation Matrix (Version 0.84).* https://github.com/taiyun/corrplot (accessed October 16, 2017).

[B53] Wickham H. Averick M. Bryan J. Chang W. McGowan L. François R. (2019). Welcome to the Tidyverse. *JOSS* 4:1686. 10.21105/joss.01686

[B54] Williams S. J. Jones J. P. G. Clubbe C. Sharrock S. Gibbons J. M. (2011). Why are some biodiversity policies implemented and others ignored? Lessons from the uptake of the Global Strategy for Plant Conservation by botanic gardens. *Biodivers. Conserv.* 21 175–187. 10.1007/s10531-011-0174-1

[B55] Wu R. Zhang S. Yu D. W. Zhao P. Li X. Wang L. (2011). Effectiveness of China’s nature reserves in representing ecological diversity. *Front. Ecol. Environ.* 9:383–389. 10.1890/100093

[B56] Wu Z. (1980). *The Vegetation of China.* Beijing: Science Press.

[B57] Xi J. (2021). Ecological civilization: Building a shared future for all life on earth. *BRIQ*, 2, 27–30. 10.1093/nsr/nwaa279 PMC8310764 34691691

[B58] Xie D. Liu B. Zhao L. N. Pandey T. R. Liu H. Y. Shan Z. J. (2021). Diversity of higher plants in China. *J. Syst. Evol.* 59 1111–1123. 10.1111/jse.12758

[B59] Xu Y. Shen Z. Ying L. Wang Z. Huang J. Zang R. (2017). Hotspot analyses indicate significant conservation gaps for evergreen broadleaved woody plants in China. *Sci. Rep.* 7:1859. 10.1038/s41598-017-02098-0 28500284 PMC5431964

[B60] Yang X. Liu B. Bussmann R. W. Guan X. Xu W. Xue T. (2021). Integrated plant diversity hotspots and long-term stable conservation strategies in the unique karst area of southern China under global climate change. *For. Ecol. Manag.* 498:119540. 10.1016/j.foreco.2021.119540

[B61] Zhang D. Ye J. Su H. (2016). Quantitative approaches to identify floristic units and centres of species endemism in the Qinghai-Tibetan Plateau, south-western China. *J. Biogeogr.* 43 2465–2476. 10.1111/jbi.12819

[B62] Zhang H. N. Qin W. H. Li Z. L. Xu W. G. Xia X. Jiang M. K. (2016). Evaluation of In-situ Conservation of Higher Plants in China. *J. Ecol. Rural Environ.* 32 1–6.

[B63] Zhang L. Luo Z. Mallon D. Li C. Jiang Z. (2017). Biodiversity conservation status in China’s growing protected areas. *Biol. Conserv.* 210 89–100. 10.1016/j.biocon.2016.05.005

[B64] Zhang Y. Wang Y. Phillips N. Ma K. Li J. Wang W. (2017). Integrated maps of biodiversity in the Qinling Mountains of China for expanding protected areas. *Biol. Conserv.* 210 64–71. 10.1016/j.biocon.2016.04.022

[B65] Zhang Y. Ma K. (2008). Geographic distribution patterns and status assessment of threatened plants in China. *Biodivers. Conserv.* 17 1783–1798. 10.1007/s10531-008-9384-6

[B66] Zhang Y. Wang G. Zhuang H. Wang L. Innes J. L. Ma K. (2021). Integrating hotspots for endemic, threatened and rare species supports the identification of priority areas for vascular plants in SW China. *For. Ecol. Manag.* 484:118952. 10.1016/j.foreco.2021.118952

[B67] Zhang Z. He J. Li J. Tang Z. (2015a). Distribution and conservation of threatened plants in China. *Biol. Conserv.* 192 454–460. 10.1016/j.biocon.2015.10.019

[B68] Zhang Z. Yan Y. Tian Y. Li J. He J.-S. Tang Z. (2015b). Distribution and conservation of orchid species richness in China. *Biol. Conserv.* 181 64–72. 10.1016/j.biocon.2014.10.026

[B69] Zhao L. Li J. Liu H. Qin H. (2016). Distribution, congruence and hotspots of higher plants in China. *Sci. Rep.* 6:19080. 10.1038/srep19080 26750244 PMC4707485

[B70] Zheng H. Gao J. Xie G. Zou C. Jin Y. (2019). Ecological Corridor. *J. Ecol. Rural Environ.* 35 137–144. 10.3897/natureconservation.27.23728

[B71] Zhou D. Q. Grumbine R. E. (2011). National parks in China: Experiments with protecting nature and human livelihoods in Yunnan province, Peoples’. Republic of China (PRC). *Biol. Conserv.* 144 1314–1321. 10.1016/j.biocon.2011.01.002

[B72] Zhu Z. X. Harris A. J. Nizamani M. M. Thornhill A. H. Scherson R. A. Wang H. F. (2021). Spatial phylogenetics of the native woody plant species in Hainan. *China. Ecol. Evol.* 11 2100–2109. 10.1002/ece3.7180 33717445 PMC7920777

