# Supplementary material for: Biodiversity priority areas and conservation strategies for seed plants in China
Source: Front Plant Sci. 2022 Aug 12;13:962609. doi: 10.3389/fpls.2022.962609 (PMC9412182; doi:10.3389/fpls.2022.962609)
Supplement: Supplementary file 1 [file Data_Sheet_1.doc]

**Supplementary Figures S1-S7**

**
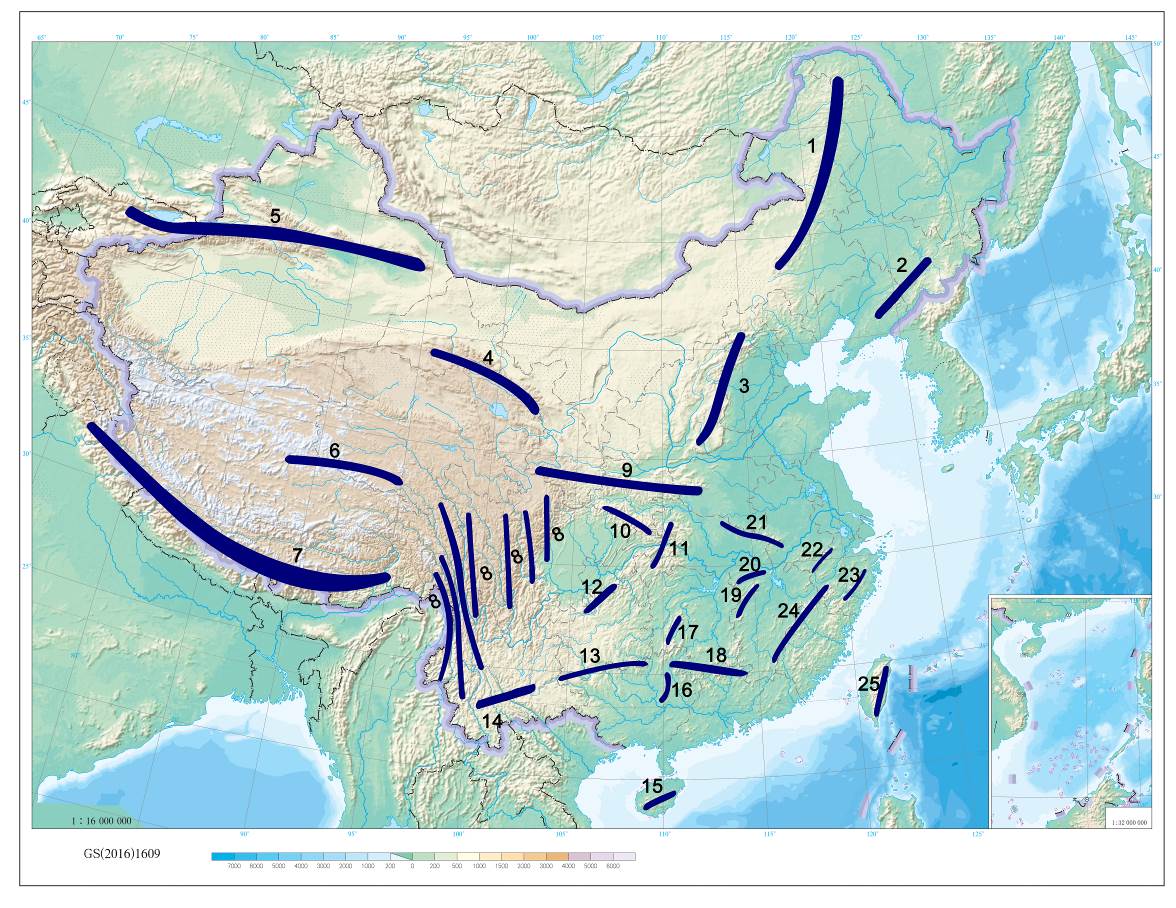
Figure S1** Major mountain ranges in China involved in this study. Black number indicates main mountain ranges: 1 Da Hinggan Range, 2 Changbai Mountain, 3 Taihang Mountain, 4 Qilian Mountain, 5 Tianshan Mountains, 6 Tanggula Mountains, 7 Himalayas, 8 Hengduan Mountains, 9 Qinling Mountains, 10 Daba Mountain, 11 Wushan Mountain, 12 Daloushan Mountain, 13 mountainous area of Guizhou and Guangxi, 14 Yunnan Plateau, 15 mountainous area of Hainan, 16 Daloushan Mountain, 17 Xuefeng Mountain, 18 Nanling Mountains, 19-20 Mufu-Lianyun-Jiuling Mountains, 21 Dabie Mountain, 22 Huangshan, 23 Tianmu Mountains, 24 Wuyi Moutain, 25 mountainous area of Taiwan.

**
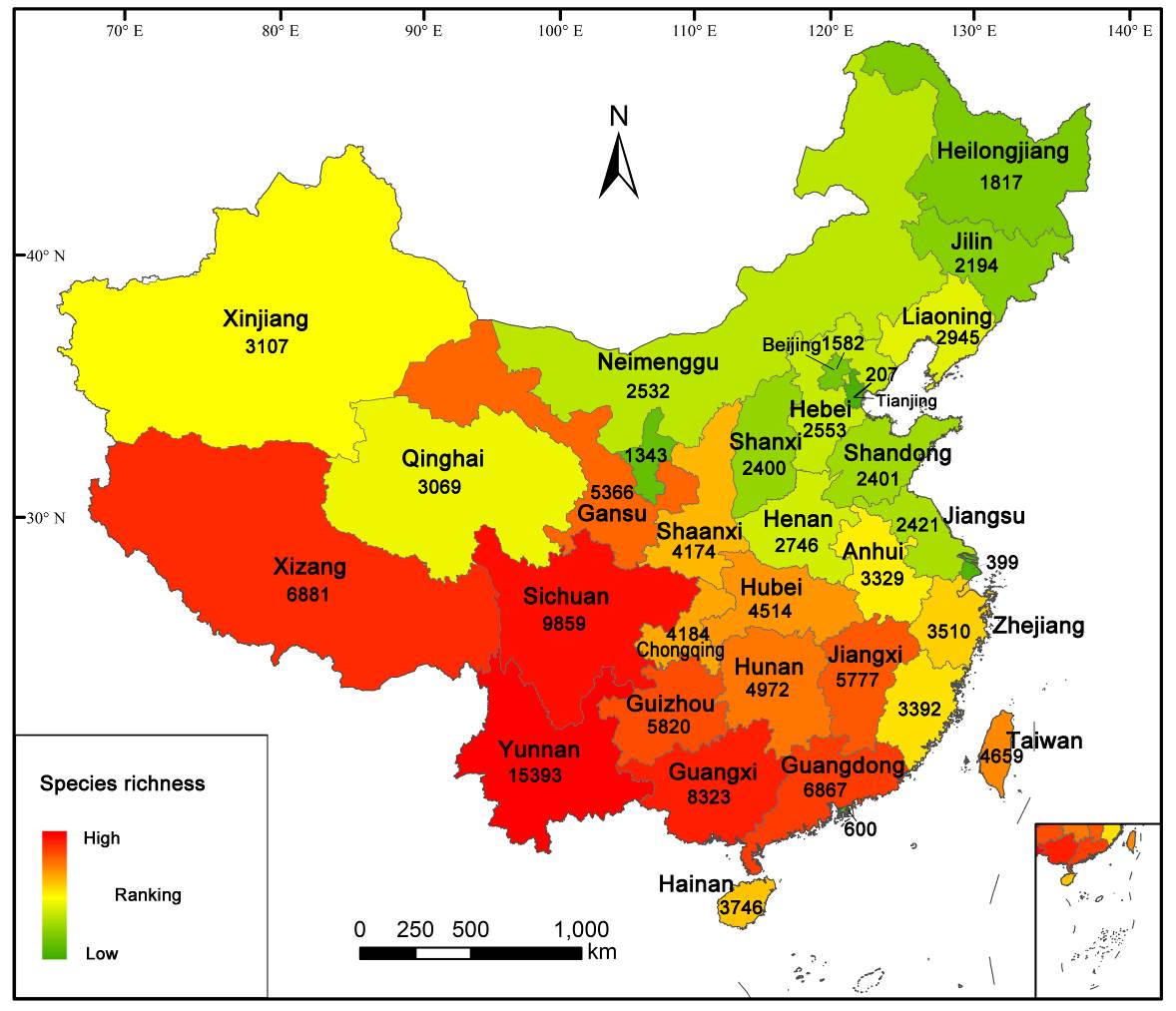
Figure S2** Species richness of administrative provinces in China.

**
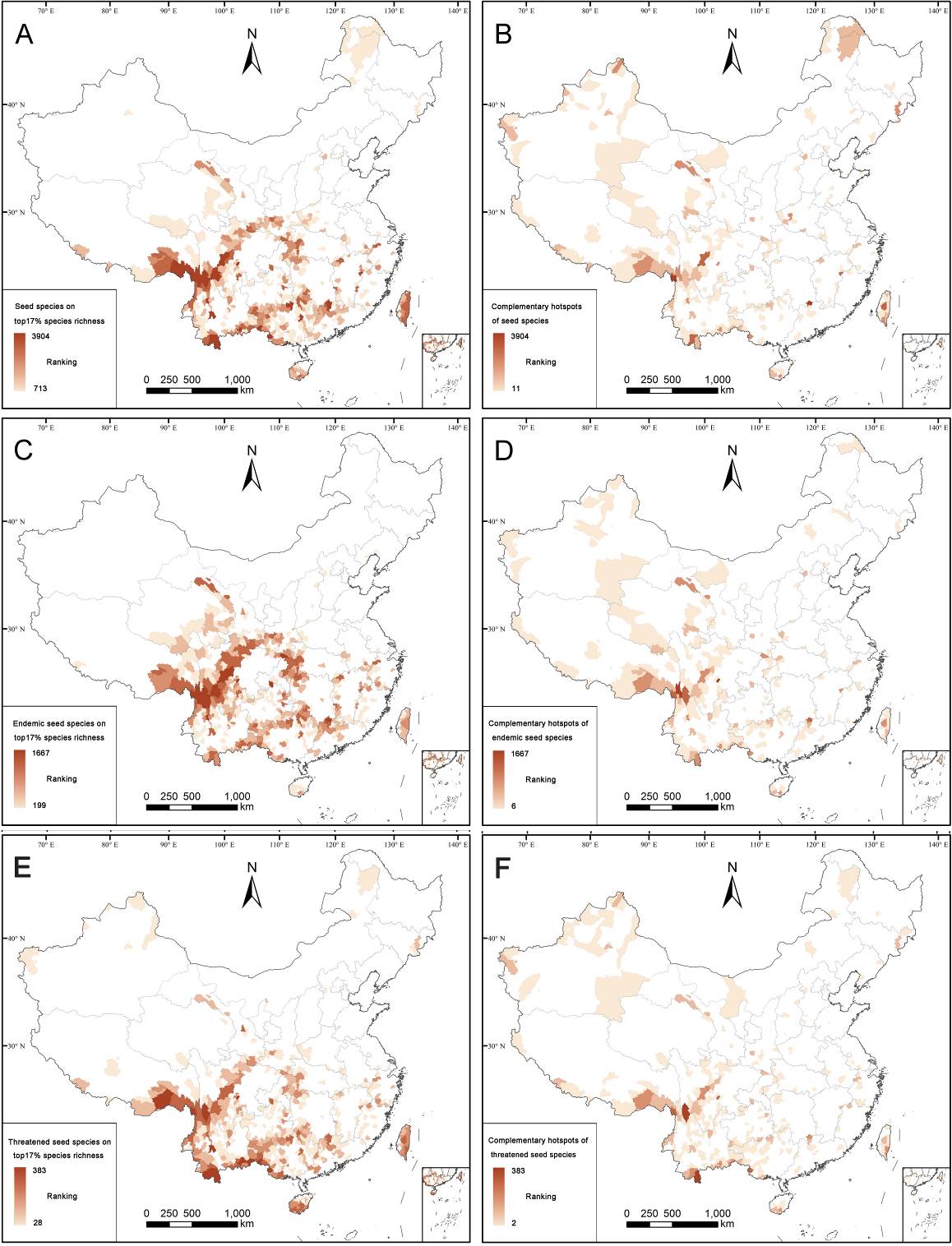
Figure S3** Distribution patterns of the species richness algorithm and complementary algorithm for all, endemic and threatened seed species with the counties which have the greatest number of species and the closest to 17% of land area.


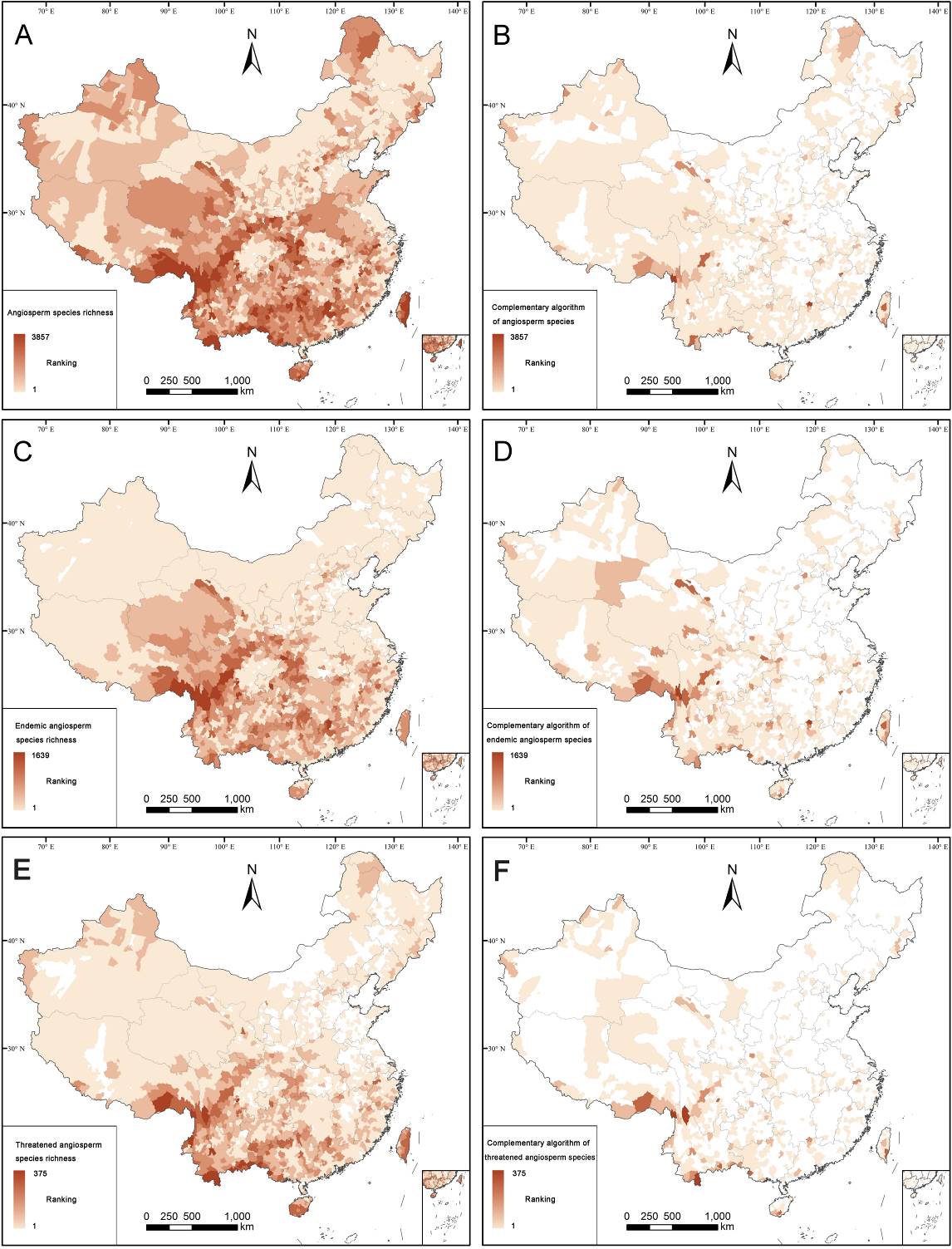
**Figure S4** Distribution patterns of the species richness algorithm and complementary algorithm for angiosperms, endemic and threatened angiosperms.


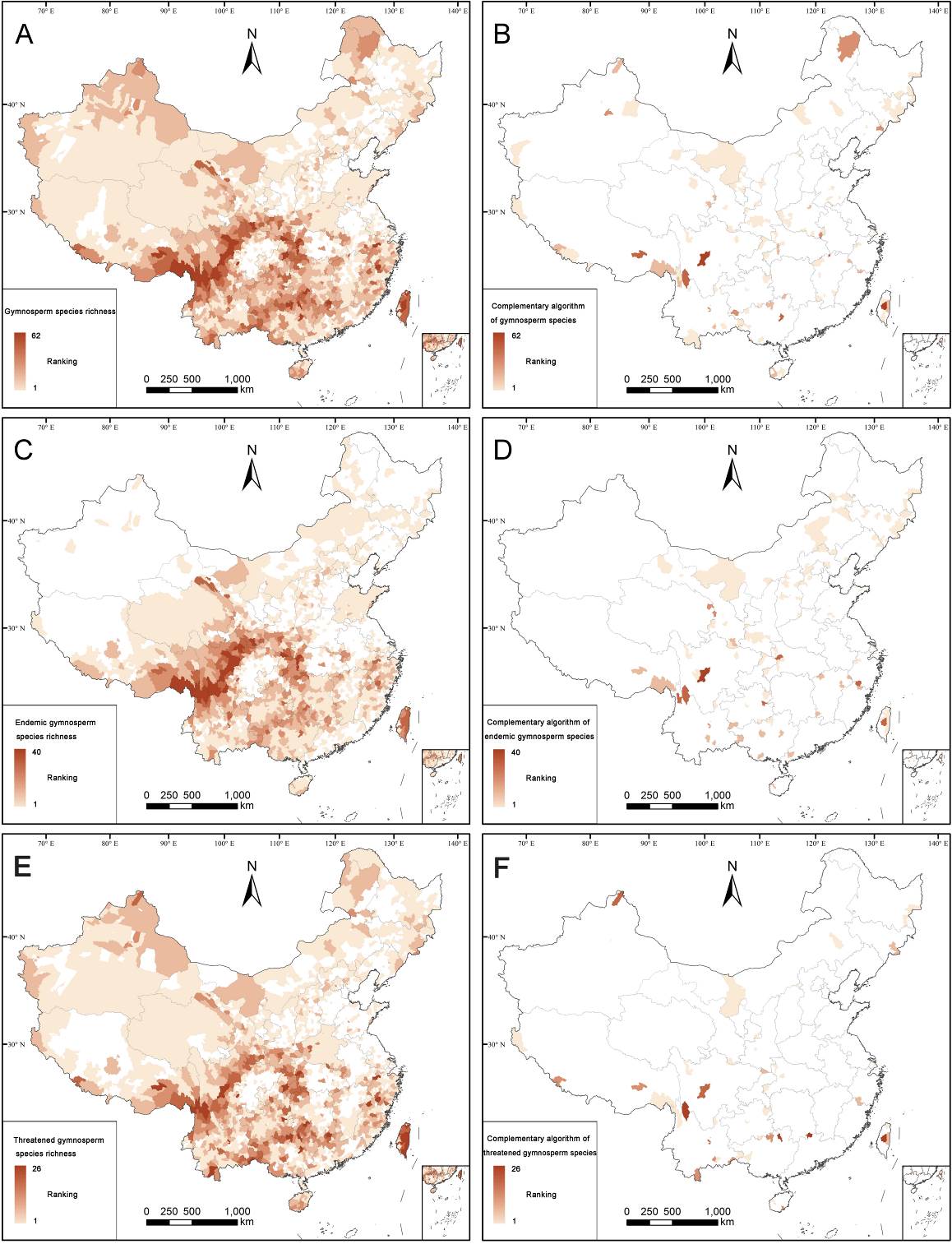
**Figure S5** Distribution patterns of the species richness algorithm and complementary algorithm for gymnosperms, endemic and threatened gymnosperms.


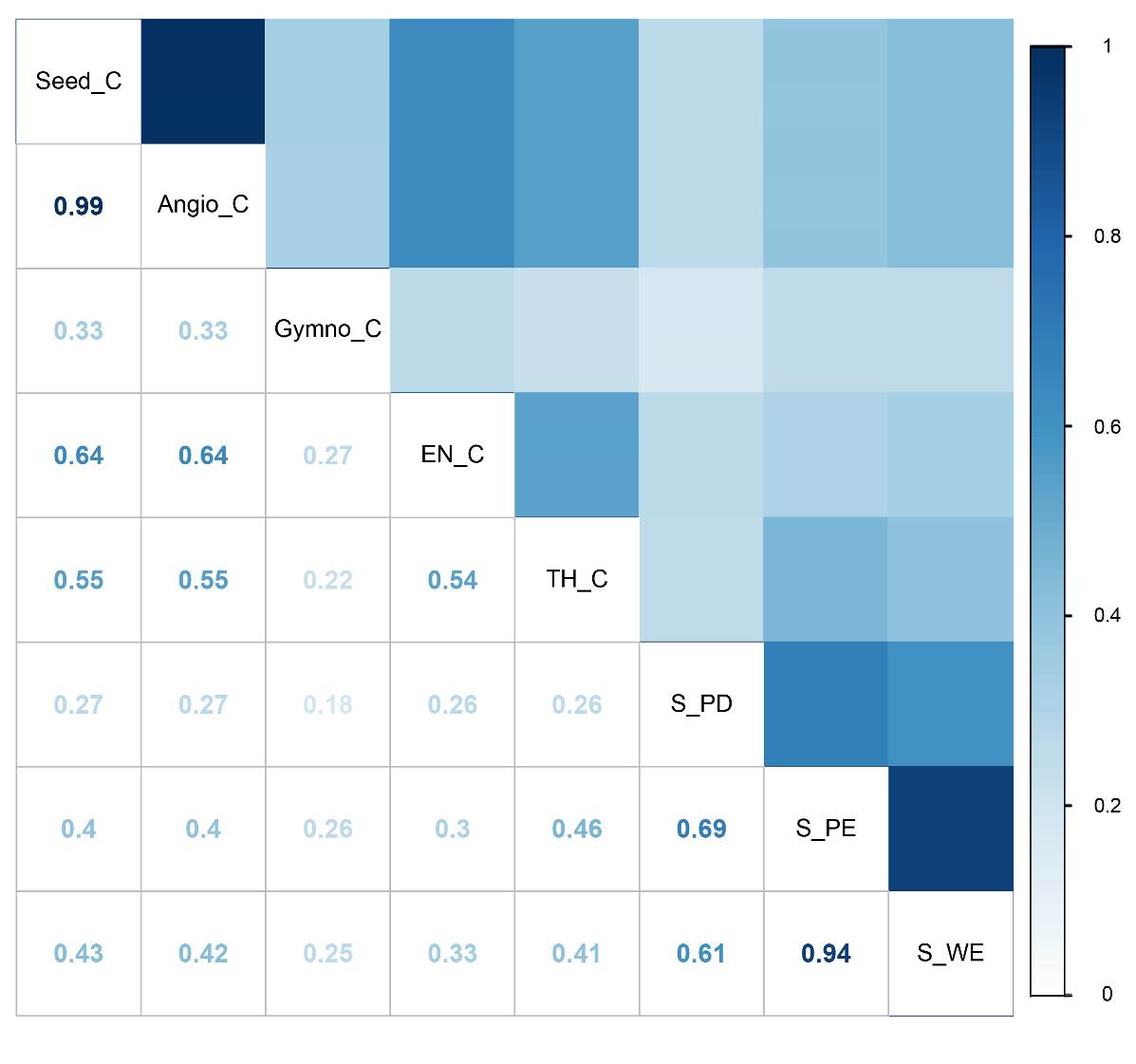
**Figure S6** The correlation analysis of the complementary algorithm for seed species, angiosperms, gymnosperms, endemic and threatened species, and the spatial phylogenetics for PD, PE and WE.

**
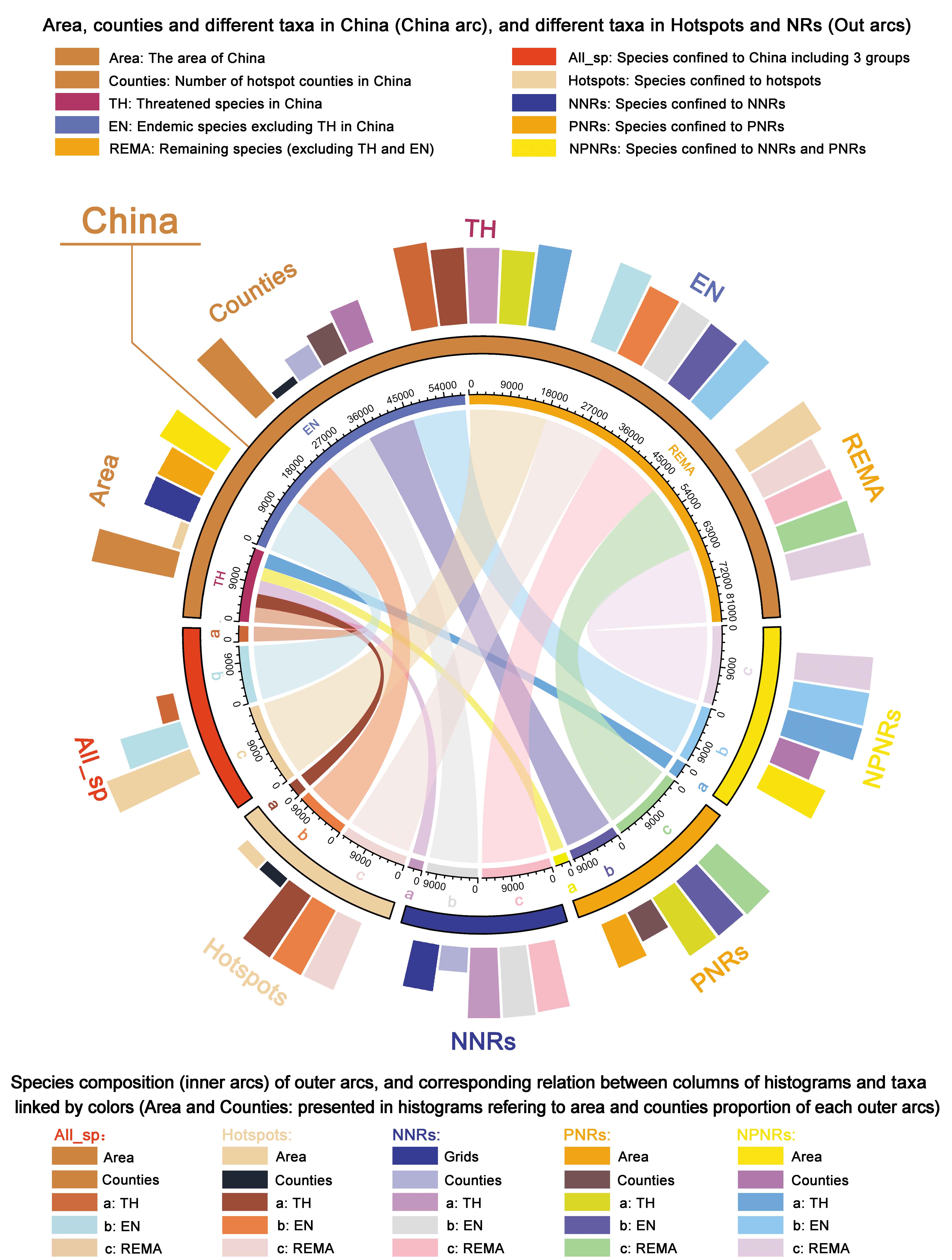
Figure S7** Chord diagram and circular barplot showing the connections between the number and area of counties and species composition of seed plants in hotspots and conservation networks. The inner arcs are connected to the circular barplot of the same color to represent the same group. The colored segments in the inner arc represent the number of species of a certain taxonomic group, or different species numbers of a certain taxonomic group in different areas. The part of all species (All_sp) presents species composition of seed plants in China in inner segments and the proportion of different taxa in the histogram.
