# Supplementary material for: Biodiversity priority areas and conservation strategies for seed plants in China
Source: Front Plant Sci. 2022 Aug 12;13:962609. doi: 10.3389/fpls.2022.962609 (PMC9412182; doi:10.3389/fpls.2022.962609)
Supplement: Supplementary file 3 [file Data_Sheet_3.doc]

**Supplementary Tables S2.1-S2.5**

**Table S2.1 Species statistics of hotspot counties with 17% of land area and highest values identified by the species richness algorithm, complementary algorithm and spatial phylogenetics, and overlapped biodiversity hotspots of three algorithms.**

|  | | All seed species | All angiosperm species | All gymnosperm species | Families of all species | Genera of all species | Endemic species | Endangered species |
| --- | --- | --- | --- | --- | --- | --- | --- | --- |
| Seed plants | Species richness algorithm hotspots | 30282/88.85% | 29967/88.87% | 315/87.26% | 264/98.14% | 2931/93.25% | 14016/93.07% | 3274/93.25% |
|  | Complementary algorithm hotspots | 31764/93.20% | 31420/93.18% | 344/95.29% | 266/98.88% | 3058/97.30% | 13854/91.99% | 3309/94.25% |
|  | Spatial phylogenetics hotspots | 31502/92.43% | 31155/92.39% | 347/96.12% | 265/98.51% | 3056/97.23% | 13759/91.36% | 3295/93.85% |
|  | Overlapped biodiversity hotspots | 31637/92.83% | 31296/92.81% | 341/94.46% | 265/98.51% | 3044/96.85% | 14020/93.09% | 3311/94.30% |

**Table S2.2** Statistics of species number, hotspot counties, conservation effectiveness and gaps of the top 5%, top 10% and top 17% biodiversity hotspots for seed species.

| Hotspots | Plant taxa and species number | | | Conservation effectiveness | | | Conservation gaps | | |
| --- | --- | --- | --- | --- | --- | --- | --- | --- | --- |
|  | All species | Endemic species | Threatened species | NNRs | PNRs | NNRs & PNRs | NNRs | PNRs | NNRs & PNRs |
| Top 5% biodiversity hotspots  (110 hotspot counties) | 26189/76.84% | 12080/80.21% | 2868/81.69% | 23570/69.16%/  (86 counties) | 23124/67.85%/  (75 counties) | 26030/76.37%/  (107 counties) | 13700/40.20%/  (24 counties) | 18337/53.80%/  (35 counties) | 3525/10.34%/  (3 counties) |
| Top 10% biodiversity hotpots  (264 hotspot counties) | 29045/85.22% | 13248/87.97% | 3164/90.12% | 25912/76.03%/  (181 counties) | 25848/75.84%/  (177 counties) | 28584/83.87%/  (239 counties) | 19009/55.77%/  (83 counties) | 20972/61.53%/  (87 counties) | 9862/28.94%/  (25 counties) |
| Top 17% biodiversity hotspots  (397 hotspot counties) | 31637/92.83% | 14020/93.09% | 3311/94.30% | 27948/82%/  (257 counties) | 27613/81.02%/  (247 counties) | 30828/90.45%/  (345 counties) | 22071/64.76%/  (140 counties) | 24377/71.52%/  (150 counties) | 13391/39.29%/  (52 counties) |

**Table S2.3** The ultimate biodiversity hotspot areas and their identification in the three algorithms for seed species. “+ + +” represents the main distribution area; “+ +” represents the medium distribution area; “+” represents the minor distribution area; “-” represents almost not distribution area.

| Ultimate Hotspot areas | Species richness algorithm | | | | Complementary algorithm | | | | Spatial phylogenetics | | | |
| --- | --- | --- | --- | --- | --- | --- | --- | --- | --- | --- | --- | --- |
|  | Aggregate | Endemic | Threatened | Integrated hotspots | Aggregate | Endemic | Threatened | Integrated hotspots | PD | PE | WE | Integrated hotspots |
| Nielamu region | +++ | +++ | +++ | +++ | +++ | +++ | +++ | +++ | + | +++ | +++ | +++ |
| Southeastern part of Xizang | +++ | +++ | +++ | +++ | +++ | +++ | +++ | +++ | +++ | +++ | +++ | +++ |
| Hengduan Mountains | +++ | +++ | +++ | +++ | +++ | +++ | +++ | +++ | +++ | +++ | +++ | +++ |
| the border areas of Yunnan | +++ | +++ | +++ | +++ | +++ | +++ | +++ | +++ | +++ | +++ | +++ | +++ |
| the border areas of Guangxi | +++ | +++ | +++ | +++ | +++ | +++ | +++ | +++ | +++ | +++ | +++ | +++ |
| the boundary areas of Guizhou and Guangxi | +++ | +++ | +++ | +++ | +++ | +++ | +++ | +++ | +++ | +++ | ++ | +++ |
| Dayao Mountain | +++ | +++ | +++ | +++ | + | + | + | + | +++ | +++ | ++ | +++ |
| eastern part of Nanling Mountain | +++ | +++ | +++ | +++ | ++ | ++ | ++ | ++ | +++ | +++ | +++ | +++ |
| western part of Nanling Mountain | +++ | +++ | +++ | +++ | + | + | ++ | ++ | +++ | +++ | ++ | +++ |
| southern part of Xuefeng Mountain | +++ | +++ | +++ | +++ | + | ++ | ++ | ++ | +++ | +++ | + | +++ |
| Mufu-Lianyun- Jiuling Mountains | +++ | +++ | +++ | +++ | + | + | - | + | +++ | +++ | +++ | +++ |
| Luoxiao Mountain | +++ | +++ | +++ | +++ | + | + | + | + | +++ | +++ | +++ | +++ |
| southern part of Hainan | +++ | +++ | +++ | +++ | +++ | +++ | +++ | +++ | +++ | +++ | +++ | +++ |
| Taiwan Island | +++ | +++ | +++ | +++ | +++ | +++ | +++ | +++ | +++ | +++ | +++ | +++ |
| Wuyi Moutain | +++ | +++ | +++ | +++ | ++ | ++ | ++ | ++ | +++ | +++ | +++ | +++ |
| Huangshan-Tianmu Mountains | +++ | +++ | +++ | +++ | ++ | ++ | ++ | ++ | +++ | +++ | ++ | +++ |
| Dabie Mountain | +++ | +++ | +++ | +++ | ++ | ++ | ++ | ++ | +++ | +++ | ++ | +++ |
| Qinling Mountains | +++ | +++ | +++ | +++ | ++ | ++ | ++ | ++ | +++ | +++ | + | +++ |
| Daba-Wushan Mountains | +++ | +++ | +++ | +++ | ++ | ++ | ++ | ++ | +++ | +++ | ++ | +++ |
| Wuling Mountain | +++ | +++ | ++ | +++ | + | + | + | + | +++ | + | + | +++ |
| northern part of Qilian Mountain | +++ | +++ | +++ | +++ | +++ | +++ | +++ | +++ | +++ | +++ | +++ | +++ |
| northern part of Changbai Mountain | +++ | + | +++ | +++ | +++ | +++ | +++ | +++ | +++ | +++ | +++ | +++ |

**Table S2.4** Statistics of species number, conservation effectiveness and gaps of ultimate biodiversity hotspots for seed species.

| **Number of**  **species**  **Taxa** | | **Species number** | | **Conservation effectiveness of hotspot** | | | **Species number of conservation network** | | | **Species distributed in conservation gaps** | | |
| --- | --- | --- | --- | --- | --- | --- | --- | --- | --- | --- | --- | --- |
|  |  | **China** | **Hotspots** | **NNRs** | **PNRs** | **NNRs & PNRs** | **NNRs** | **PNRs** | **NNRs & PNRs** | **NNRs** | **PNRs** | **NNRs & PNRs** |
| **Seed species** | **All species** | 34082 | 29045 | 25912 | 25848 | 28584 | 29828 | 30300 | 33053 | 19009 | 20972 | 9862 |
|  | **Endemic species** | 15060 | 13248 | 12079 | 11515 | 13058 | 13448 | 13097 | 14631 | 8031 | 9441 | 3891 |
|  | **Endangered species** | 3511 | 3164 | 2887 | 2762 | 3124 | 3138 | 3072 | 3406 | 1772 | 2265 | 766 |
|  | **Angiosperm** | 33721 | 31296 | 25645 | 25572 | 28286 | 29522 | 29974 | 32707 | 18784 | 20736 | 9712 |
|  | **Gymnosperm** | 361 | 341 | 267 | 276 | 298 | 306 | 326 | 346 | 225 | 236 | 150 |

**Table S2.5** Species composition of threatened species (TH), endemic species excluding threatened species (EN), and rest species excluding threatened and endemic species (REST) for seed species in China, hotspots, conservation network, conservation effectiveness and gaps.

| Taxonomic groups, area and counties | Species number of China | Species number of hotspots | Species number of NNRs | Species number of PNRs | Species number of NNRs and PNRs | Conservation effectiveness of NNRs | Conservation effectiveness of PNRs | Conservation effectiveness of NNRs and PNRs | Conservation gaps of NNRs | Conservation gaps of PNRs | Conservation gaps of NNRs and PNRs |
| --- | --- | --- | --- | --- | --- | --- | --- | --- | --- | --- | --- |
| TH | 3511 | 3164 | 3138 | 3072 | 3406 | 2887 | 2762 | 3124 | 1772 | 2265 | 766 |
| EN | 12677 | 11113 | 11342 | 11037 | 12331 | 10146 | 9673 | 10957 | 6907 | 7996 | 3420 |
| REST | 17894 | 14768 | 15348 | 16191 | 17316 | 12879 | 13413 | 14503 | 10330 | 10711 | 5676 |
| Total species | 34082 | 29045 | 29828 | 30300 | 33053 | 25912 | 25848 | 28584 | 19009 | 20972 | 9862 |
| Area | 100% | 10.00% | 58.60% | 59.34% | 79.22% | 7.54% | 7.13% | 9.43% | 2.40% | 2.81% | 0.51% |
| counties | 2906 | 264 | 901 | 1182 | 1560 | 181 | 177 | 239 | 83 | 87 | 25 |
